# Induction of Antibacterial Metabolites by Co-Cultivation of Two Red-Sea-Sponge-Associated Actinomycetes *Micromonospora* sp. UR56 and *Actinokinespora* sp. EG49

**DOI:** 10.3390/md18050243

**Published:** 2020-05-05

**Authors:** Mohamed S. Hifnawy, Hossam M. Hassan, Rabab Mohammed, Mohamed M. Fouda, Ahmed M. Sayed, Ahmed A. Hamed, Sameh F. AbouZid, Mostafa E. Rateb, Hani A. Alhadrami, Usama Ramadan Abdelmohsen

**Affiliations:** 1 Department of Pharmacognosy, Faculty of Pharmacy, Cairo University, 11787 Cairo, Egypt; mhifnawy@hotmail.com; 2 Department of Pharmacognosy, Faculty of Pharmacy, Beni-Suef University, 62514 Beni-Suef, Egypt; abuh20050@yahoo.com (H.M.H.); rmwork06@yahoo.com (R.M.); sameh.zaid@pharm.bsu.edu.eg (S.F.A.); 3 Department of Pharmacognosy, Faculty of Pharmacy, Nahda University, 62513 Beni-Suef, Egypt; foudapharma@gmail.com (M.M.F.); ahmed.mohamed.sayed@nub.edu.eg (A.M.S.); 4 Microbial Chemistry Department, National Research Center, 33 El-Buhouth Street, 12622 Giza, Egypt; ahmedshalbio@gmail.com; 5 School of Computing, Engineering & Physical Sciences, University of the West of Scotland, Paisley PA1 2BE, UK; mostafa.rateb@uws.ac.uk; 6 Department of Medical Laboratory Technology, Faculty of Applied Medical Sciences, King Abdulaziz University, Jeddah 21589, Saudi Arabia; 7 Special Infectious Agent Unit, King Fahd Medical Research Centre, King Abdulaziz University, Jeddah 21589, Saudi Arabia; 8 Department of Pharmacognosy, Faculty of Pharmacy, Minia University, 61519 Minia, Egypt; 9 Department of Pharmacognosy, Faculty of Pharmacy, Deraya University, Universities Zone, P.O. Box 61111 New Minia City, Egypt

**Keywords:** co-cultivation, phenazine, sponge-associated actinomycetes, antibacterial, antibiofilm, DNA gyrase, pyruvate kinase

## Abstract

Liquid chromatography coupled with high resolution mass spectrometry (LC-HRESMS)-assisted metabolomic profiling of two sponge-associated actinomycetes, *Micromonospora* sp. UR56 and *Actinokineospora* sp. EG49, revealed that the co-culture of these two actinomycetes induced the accumulation of metabolites that were not traced in their axenic cultures. Dereplication suggested that phenazine-derived compounds were the main induced metabolites. Hence, following large-scale co-fermentation, the major induced metabolites were isolated and structurally characterized as the already known dimethyl phenazine-1,6-dicarboxylate (**1**), phenazine-1,6-dicarboxylic acid mono methyl ester (phencomycin; **2**), phenazine-1-carboxylic acid (tubermycin; **3**), N-(2-hydroxyphenyl)-acetamide (**9**), and *p*-anisamide (**10**). Subsequently, the antibacterial, antibiofilm, and cytotoxic properties of these metabolites (**1**–**3**, **9**, and **10**) were determined in vitro. All the tested compounds except 9 showed high to moderate antibacterial and antibiofilm activities, whereas their cytotoxic effects were modest. Testing against *Staphylococcus* DNA gyrase-B and pyruvate kinase as possible molecular targets together with binding mode studies showed that compounds **1**–**3** could exert their bacterial inhibitory activities through the inhibition of both enzymes. Moreover, their structural differences, particularly the substitution at C-1 and C-6, played a crucial role in the determination of their inhibitory spectra and potency. In conclusion, the present study highlighted that microbial co-cultivation is an efficient tool for the discovery of new antimicrobial candidates and indicated phenazines as potential lead compounds for further development as antibiotic scaffold.

## 1. Introduction

Natural products derived from marine microbes play a pivotal role in drug discovery and development due to their diverse molecular and chemical scaffolds, which cannot be matched by any synthetic or combinatorial libraries [1]. Marine-sponge-associated actinomycetes have recently been used to produce a multitude of new bioactive compounds with novel molecular scaffolds and potent pharmacological activities such as antibacterial, antifungal, antiparasitic, immunomodulatory, anti-inflammatory, antioxidant, and anticancer activities [2,3,4,5]. However, searching for new and promising bioactive secondary metabolites from marine microbes is becoming a serious challenge due to the increasing rate of rediscovery of known secondary metabolites [6,7]. On the other hand, genomic work has revealed that definite groups of bacteria and fungi have huge numbers of silent biosynthetic gene clusters (BGCs) that encode for secondary metabolites that are not traced under normal laboratory conditions [8,9]. Microbial competition for space and nutrition are considered eventual routes for the induction of bioactive metabolites, mainly antimicrobial agents [10]. Several protocols have been used to trigger such cryptic biosynthetic pathways. One of these protocols involves co-cultivation or the so-called mixed fermentation of two or more microorganisms in a single culture flask, which results in the production of antimicrobial secondary metabolites [11,12,13]. Recently, we reported the production of new antitumor agents, saccharomonosporine A and convolutamydine F, by the co-fermentation of two marine-sponge-associated actinomycetes, *Dietzia* sp. UR66 and *Saccharomonospora* sp. UR22, obtained from *Callyspongia siphonella* [14]. A chlorinated benzophenone pestalone that showed potent antibiotic activity was sourced from the co-cultivation of two marine-associated fungi, α-proteobacterium CNJ-328 and *Pestalotia* sp. CNL-365 [15]. The induction of cryptic pulicatin derivatives that exhibit potent antifungal effects through the microbial co-culture of *Pantoea agglomerans* with *Penicillium citrinum* was recently reported [16]. Finally, the induced production of emericellamides A and B obtained from a co-fermentation of the marine-associated fungus *Emericella* sp. CNL-878 and the marine derived bacterium *Salinispora arenicola* was reported [17]. 

**Figure 1:**
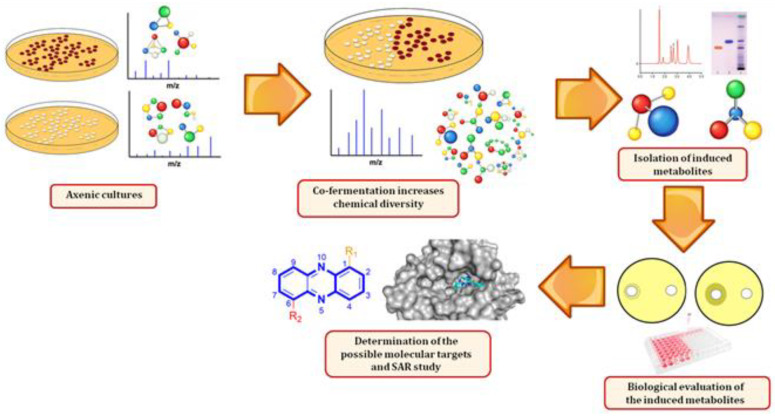
Outline of the procedure used in the present study.

Phenazine compounds are heterocyclic nitrogenous compounds that consist of two benzene rings attached through two nitrogen atoms and substituted at different sites of the core ring system. Phenazine derivatives have been found to show a wide range of biological activities, including antibacterial, antiviral, antitumor, antimalarial, and antiparasitic activities [18,19]. They have been isolated in large amounts from terrestrial bacteria such as *Pseudomonas*, *Streptomyces*, and other genera from marine habitats [20]. The iminophenazine clofazimine is an example of a successful phenazine derivative, having been approved by the FDA for the treatment of leprosy and drug-resistant *Mycobacterium tuberculosis* strains [21,22]. Another example of a phenazine is bis-(phenazine-1-carboxamide), which acts as a strong cytotoxin and represents an attractive class of anticancer drugs [23]. In an earlier work, we found that *Actinokineospora* sp. EG49 was able to induce *Nocardiopsis* sp. RV163 to produce 1,6-dihydroxyphenazine upon co-cultivation [24]. On the other hand, *Micromonospora* sp. are widespread actinomycetes and prolific producers of diverse antibiotics [25,26]. Consequently, we decided to extend our co-cultivation trials on both marine-derived *Actinokineospora* sp. EG49 and *Micromonospora* sp. UR56 in order to induce the production of further antibacterial metabolites, which were also found to be of the phenazine class. Based on earlier reports on the biological activities of this class of compounds, we suggested both DNA gyrase B (Gyr-B) and pyruvate kinase (PK) to be the possible molecular targets of their antibacterial activity. The working outline of the present study is illustrated in Figure 1.

## 2. Results and Discussion

### 2.1. Metabolomic Profiles of the Axenic and Co-Culture Extracts

**Figure 2:**
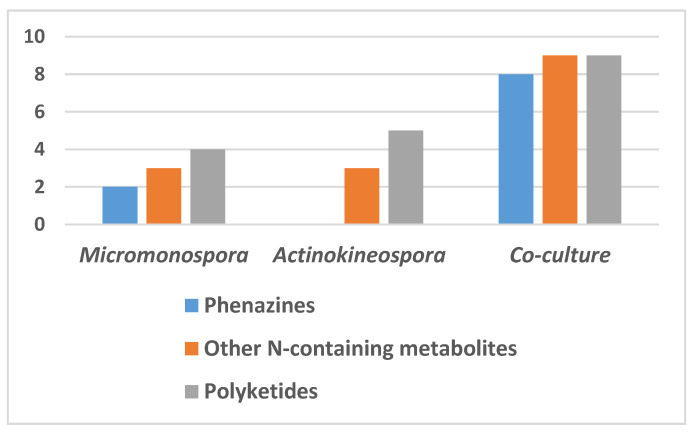
Classes of metabolites produced from *Micromonospora* sp. UR56 and *Actinokineospora* sp. EG49 axenic and co-cultures.

**Figure 3:**
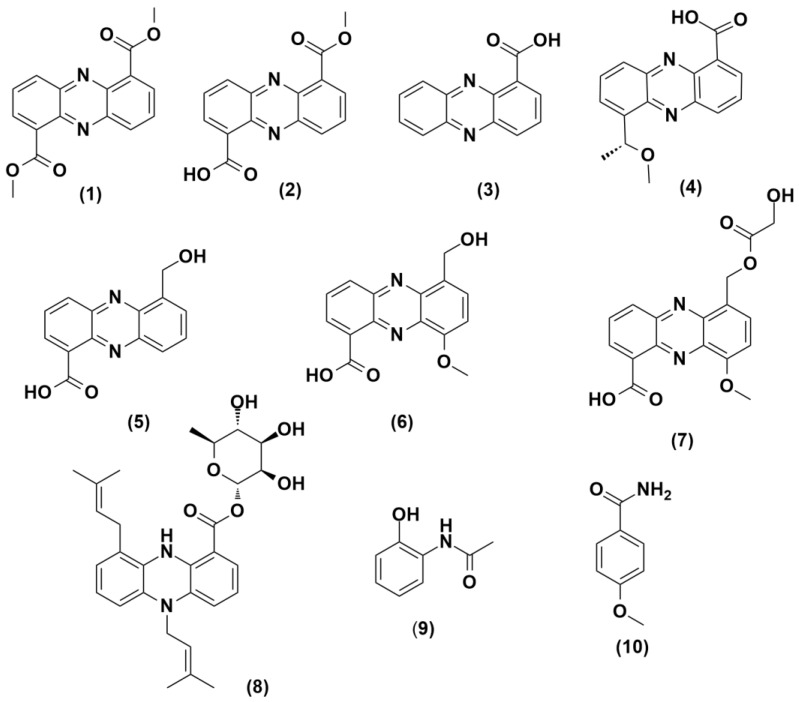
Identified phenazine derivatives in the axenic *Micromonospora* sp. UR56 culture, and after its co-culture with *Actinokineospora* sp. EG49. **1**: dimethyl phenazine-1,6-dicarboxylate, **2**: phencomycin, **3**: phenazine-1-carboxylic acid, **4**: methyl saphenate, **5**: 1-hydroxy methyl-6-carboxy phenazine, **6**: griseolutic acid, **7**: griseolutin A, **8**: aestivophoenin C, **9**: N-(2-hydroxyphenyl)-acetamide, and **10**: *p*-anisamide.

The chemical profiles of the actinomycetes *Micromonospora* sp. UR56 and *Actinokineospora* sp. EG49 were investigated via liquid chromatography coupled with mass spectrometry (LC-HRMS) analysis after their fermentations (axenic and co-fermentation). The metabolomic profile of the co-culture extract displayed the induction of diverse metabolites from different chemical classes compared to those of the two axenic cultures (Figure 2, Supplementary Figure S32, and Supplementary Table S3). Twelve metabolites were putatively identified in the *Micromonospora* sp. UR56-derived extract, where phenazine derivatives were found to prevail (Figure 2; Figure 3, Supplementary Figure S30). Most of these dereplicated phenazines e.g., phenazine-1-carboxylic acid (**3**), aestivophoenin c (**8**), and methyl saphenate (**4**) have been reported to possess antimicrobial and cytotoxic properties [27]. The remaining identified compounds were found to belong to the N-containing and polyketide classes. Within the axenic *Actinokineospora* sp. EG49 culture, no phenazine derivatives were traced in the LC-HRMS analysis of the extract. Additionally, its chemical profile revealed poor diversity, with a few identified N-containing and polyketide metabolites (Supplementary Figure S31 and Supplementary Table S2). On the other hand, the mixed fermentation of both actinomycetes induced *Micromonospora* sp. UR56 to accumulate diverse phenazine derivatives (**1**–**8**) (Figure 2). Such induction could be due to environmental competition or chemical defense mechanisms [10]. Based on the metabolomic profiling of the co-culture, the major induced metabolites (**1**–**3**, **9**, and **10**) were targeted and isolated using Sephadex LH20 followed by silica gel column chromatography, and identified using different spectroscopic approaches. Subsequently, they were subjected to antibacterial, antibiofilm, and cytotoxicity testing.

### 2.2. Bioactivity Testing 

#### 2.2.1. Antibacterial Activity

**Figure 4:**
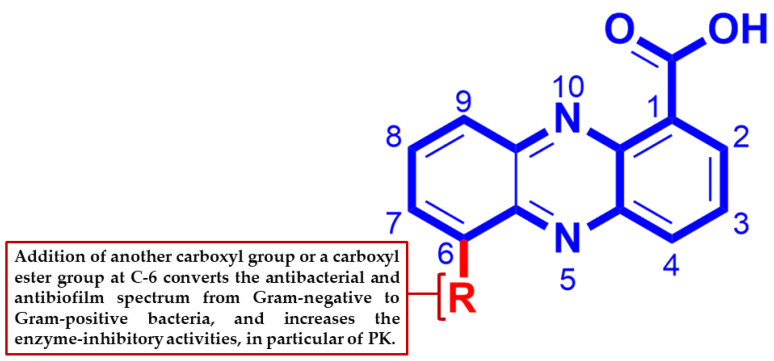
Structure–activity relationship of the induced phenazine derivatives.

Based on earlier reports on their antibacterial potential, phenazine-derived compounds have proven to be an interesting chemical scaffold for the development of new antibacterial agents [27]. Therefore, all isolated compounds were evaluated against *Staphylococcus aureus* ATCC9144, *Bacillus subtilis* ATCC29212, *Pseudomonas aeruginosa* ATCC27853, and *Escherichia coli* ATCC25922 (Table 1). Compounds **3** and **10** displayed potent antibacterial activity against *P. aeruginosa* with growth inhibition of 94% and 70%, respectively, while compounds **1**, **2**, and **9** showed considerable antibacterial activity against *S. aureus* with growth inhibition of 47%, 69%, and 53%, respectively (Table 1). Based on these results together those previously reported [27], we concluded that the phenazine-1-carboxylic acid scaffold is essential for antibacterial activity against Gram-negative bacteria and produces no observable activity towards Gram-positive ones. Although the addition of another carboxylic acid or carboxyl ester at C-6 significantly decreased the inhibitory activity against Gram-negative bacteria, it converted these phenazine derivatives to be active against Gram-positive strains (Figure 4).

#### 2.2.2. Antibiofilm Activity 

To further evaluate the inhibitory activities of the isolated metabolites (**1**–**3**, **9**, and **10**), their antibiofilm potentials against *S. aureus*, *B. subtilis*, *E. coli*, and *P. aeruginosa* were determined. Compounds **3** and **10** displayed potent antibiofilm activity against *P. aeruginosa* with % inhibition rates of 94% and 73%, respectively, while compounds **1**, **2**, and **9** showed mild to moderate inhibitory activity against *E. coli* with % inhibition ranges of 34–54%. Compounds **1** and **2** both showed potent to moderate inhibitory activity against *S. aureus* with % inhibition rates of 50% and 75%, respectively (Table 2). Similar to the antibacterial results, the presence of carboxylic acids on both C-1 and C-6 of the phenazine ring system decreased the antibiofilm effect towards Gram-negative strains, but made these derivatives active against Gram-positive ones, particularly, *S. aureus* (Figure 4).

#### 2.2.3. Cytotoxic Activity

The five induced compounds (**1**–**3**, **9**, and **10**) were additionally tested for their cytotoxic activity against four human cancer cell lines (Table 3). Only compound **9** was able to induce moderate cytotoxicity towards the tested cell lines, with IC_50_ values ranging from 10 to 36 µM. Regarding the activity of phenazine derivatives (**1**–**3**), compound **2** was found to be the most active against all tested cell lines. Previous studies have suggested that phenazine-related compounds can exert significant cytotoxic activities through the inhibition of topoisomerase enzymes [28,29,30].

#### 2.2.4. In Vitro Enzyme Assay

DNA gyrase is a topoisomerase-type enzyme present exclusively in bacterial cells. Inhibition of its subunit A (Gyr-A) leads to cell death by trapping the gyrase–DNA complex and preventing DNA replication. On the other hand, inhibition of Gyr-B blocks ATPase activity and thus deprives the cell of the energy source needed for DNA replication [31]. Gyr-B as a molecular target offers an opportunity to avoid cross-resistance to the well-known Gyr-A inhibitors, quinolones. Several phenazine and acridine derivatives have been reported to be topoisomerase I and II inhibitors in human cancer cells [28,29,30]. The characteristic planar structure of this class of compounds directs them to interact with the ATP-binding domains of the enzymes (ATPase pocket) [28]. On the other hand, PK is known to be a critical enzyme in catalyzing the final step of glycolysis, which involves the transfer of a phosphoryl group from phosphoenolpyruvate to ADP, producing pyruvate and ATP [32]. Recently, it was identified as a “superhub”, listed among the top 1% of all interacting proteins in *S. aureus* and found to be a key regulator of the quorum-sensing system and the biofilm formation process in staphylococci [33,34,35]. Considering these points together with the structural similarity of our compounds (**1**–**3**) to previously well-known topoisomerase inhibitors, we selected *Staphylococcus* DNA gyrase-B and PK as possible molecular targets to mediate the observed antibacterial and antibiofilm activities of the induced phenazine derivatives toward *S. aureus*. In vitro studies (Table 4) showed **1** to be the most active compound against both Gyr-B and PK, followed by **2** and **3**. These finding indicate a direct link between the substitutions on C-1 and C-6 and the Gyr-B- and PK-inhibitory activities of this class of metabolites (Figure 3). Although the enzyme-inhibitory activity of compounds **1**–**3** was convergent, and compound **1** was slightly more active than compound **2** in the enzyme assay studies, compound **3** was inactive, and **1** was less active than **2** in both inhibitory assays against *S. aureus*. These observations could be attributed to the bacterial cell wall permeability, where *S. aureus* may permit the diffusion of **2** more easily than **1** and prevent the crossing of compound **1**. In the same manner, *P. aeruginosa*’s outer membrane may be selective only for compound **3**, and hence make both other phenazines (**1** and **2**) inactive. According to a previous report [36], small, moderately lipophilic compounds can cross the Gram-positive bacterial membrane more easily. In contrast, Gram-negative bacteria, which have porins (hydrophilic channels) in their outer membranes, are more selective for hydrophilic compounds. The calculated LogP (cLogP) of each compound revealed that compound **2** had the optimum lipophilicity (cLogP = 1.9), whereas compound **1** was less lipophilic (cLogP = 1.12) and compound **3** was more lipophilic (cLogP = 2.97) Such differences in lipophilicity between compounds **1** to **3** could explain the opposite observations between enzyme inhibition and antibacterial and antibiofilm activities of compounds **1**–**3**.

### 2.3. Docking Study

**Figure 5:**
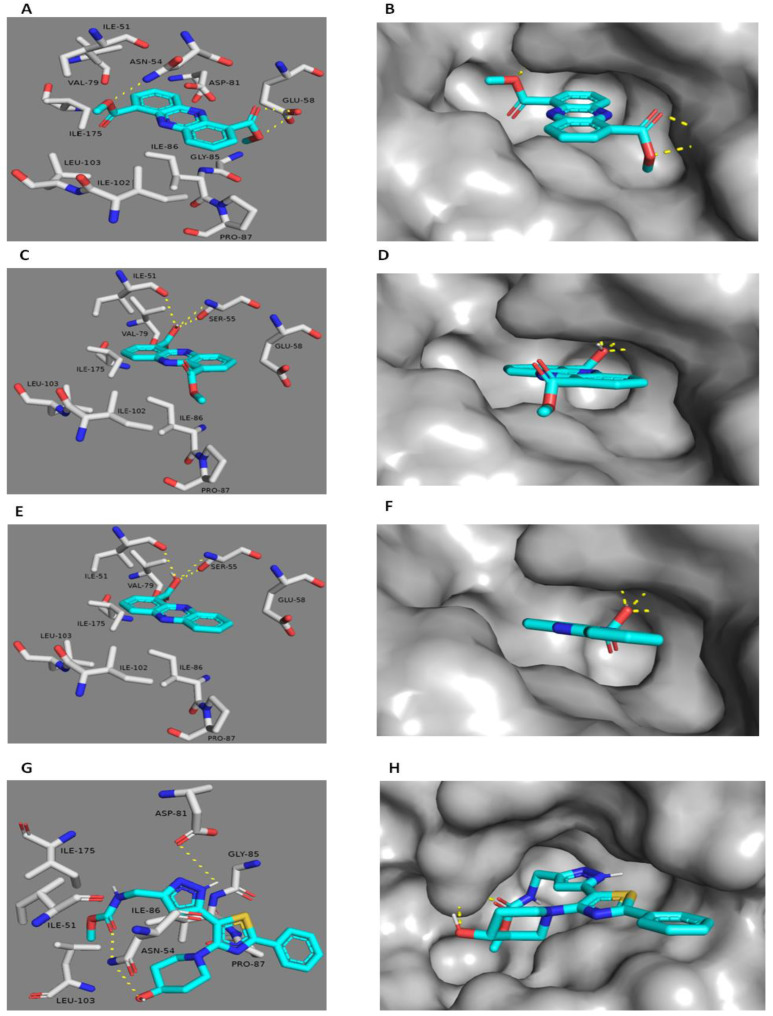
Docking of **1** (**A**,**B**), **2** (**C**,**D**) and **3** (**E**,**F**) within the active site of Staphylococcal Gyr-B. (**G**,**H**) The key binding interactions of Gyr-B co-crystallized ligand. The amino acid side chains were depicted in (**A**,**C**,**E**,**G**) for clarification.

**Figure 6:**
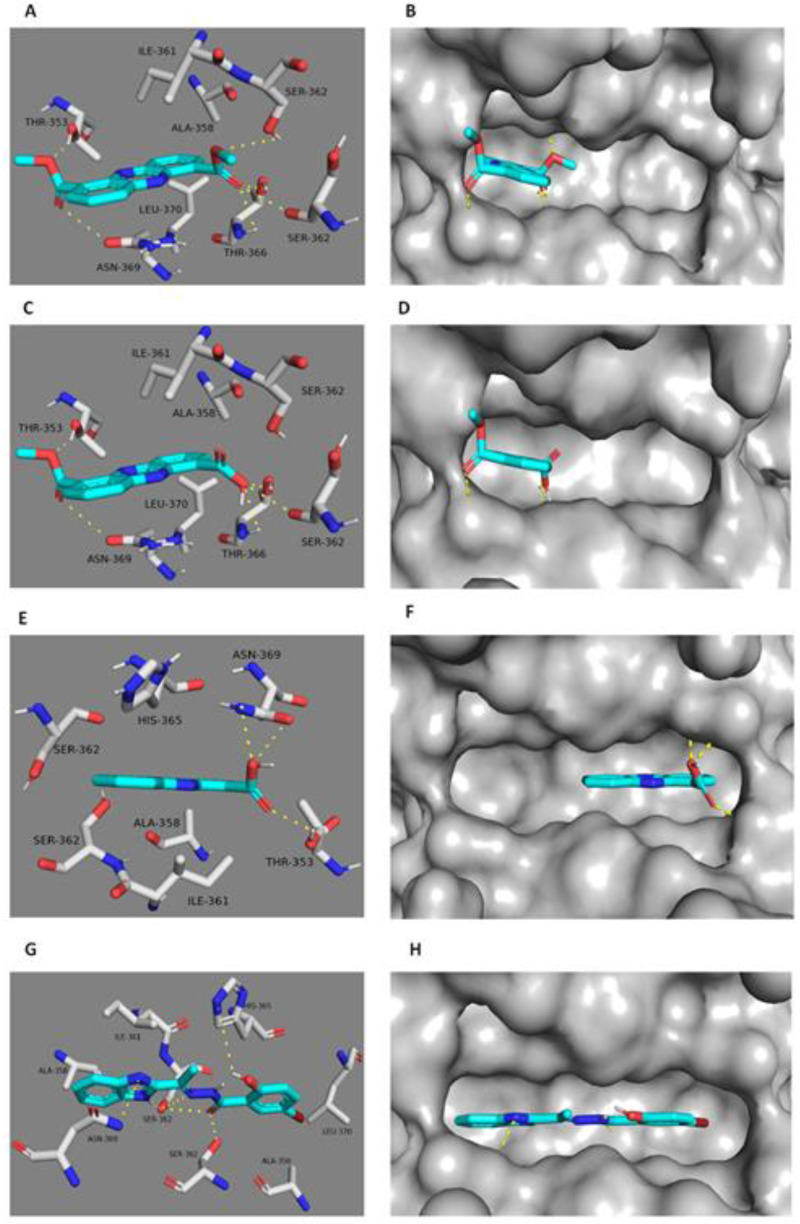
Docking of **1** (**A,B**), **2** (**C,D**), and **3** (**E,F**) within the active site of *Staphylococcus* PK. (**G,H**) The key binding interactions of the Gyr-B co-crystallized ligand. The amino acid side chains are depicted in (**A,C,E,G**) for clarification.

The potential binding modes of **1**–**3** with DNA Gyr-B and pyruvate kinase were investigated by docking at their active binding sites. *Staphylococcus* Gyr-B and PK of PDB code (3g7b and 3T0T) were selected for docking tests since they had optimum resolutions (2.3 Å and 3.1 Å) and are co-crystallized with their inhibitors. The spheres surrounding the co-crystallized inhibitors were selected as active sites for docking. All isolated compounds exhibited convergent docking poses (Figure 5) and were comparable to the co-crystallized Gyr-B inhibitor [37]. The oxygens of both ester groups of **1** on C1 and C6 were hydrogen-bonded to ASN-54, similarly to the co-crystallized Gyr-B ligand, and GLU-58. These interactions meant that the whole molecule was well-embedded inside the active site pocket, where it was further stabilized by the hydrophobic interactions between the molecule’s aromatic rings and ILE-51, VAL-79, PRO-87, ILE-86, ILE-102, ILE-103, and ILE-175. The absence of an ester group in **2** and **3** led them to take a different and less stable orientation inside the binding pocket and hence, their carboxylic acid moieties interacted from only one side with different amino acid residues via hydrogen bonds (SER-55 and ILE-51); however, they showed similar hydrophobic interactions to **1**. Unlike the co-crystallized Gyr-B inhibitor, **1**–**3** did not show any interactions with ASP-81. On the other hand, **1** and **2** showed similar binding modes in the PK active site, although different from that of **3**. The co-crystallized PK inhibitor [38] fitted perfectly inside the pocket formed between the enzyme’s A and B subunits (Figure 6). This binding mode was stabilized by six main hydrogen bonds with SER-362A, SER-362B, ASN-369B, and HIS-365A (A and B notations correspond to PK subunits). These interacting residues are of particular interest as they are not conserved in human PK, and thus are likely to further influence the selectivity of the bacterial PK inhibitors. Herein, **1** showed a convergent binding mode to the co-crystallized PK inhibitor, where it anchored on one side of the PK active site (Figure 6) through three hydrogen bonds with SER-362A and SER-362B, similarly to the co-crystallized ligand, and through an additional hydrogen bond with THR-366A. All these interactions were from the ester moiety of one side of the molecule; the second ester group on the other side increased the molecular stability inside the binding site through hydrogen bonding to THR-353B and ASN-369A. Additionally, the aromatic planar structure of **1** was sandwiched between two hydrophobic surfaces inside the binding pocket, where it interacted via hydrophobic interactions with ALA-358B and ILE-361B from one side, and with LEU-370A from the other side. Regarding compound **2**, it revealed a binding mode and interactions quite similar to that of **1**. Such strong interactions with the PK binding side explain the good in vitro inhibitory activity of both **1** and **2** (Table 4) in comparison to **3**, which showed different binding modes inside the enzyme’s binding cavity. Unlike **1** and **2**, and due to its single carboxylic acid moiety, **3** fitted inside the other corner of the active pocket through only three hydrogen bonds with ASN-369B and THR-353A. Regarding hydrophobic interactions, it interacted with only two residues, ALA-358A, and ILE-361A (Figure 6). These weaker interactions explain the inferior in vitro inhibitory activity of **3** toward PK (Table 4). In addition, it indicates the importance of the 1,6-dicarboxylic acid moieties in the future development of *Staphylococcus* PK inhibitors.

## 3. Material and Methods

### 3.1. General Experimental Procedures 

The chemical solvents used in this study, such as n-hexane, dichloromethane, ethyl acetate, and methanol, were obtained from Sigma-Aldrich, Saint Louis, Missouri, USA. Silica gel 60 (63–200 µm, E. Merck, Sigma-Aldrich) and Sephadex LH20 (0.25–0.1 mm, GE Healthcare, Sigma-Aldrich) were used for chromatographic isolation and purification. Thin-layer chromatography was performed using pre-coated silica gel aluminum plates (E. Merck, Darmstadt, Germany, Kieselgel 60 F254, 20 × 20 cm, 0.25 mm). *p*-anisaldehyde (0.5 : 85 : 10 : 5 *p-anisaldehyde* : methanol : glacial acetic acid : sulfuric acid) was used as visualizing spray reagent for different spots accompanied by heating at 110 °C. 1D, 2D, and ^13^C NMR spectra were recorded on a JEOL ECA-600 spectrometer (600 MHz for ^1^H and 150 MHz for ^13^C, respectively). Each sample was dissolved in prober deuterated solvent such as CDCl_3_ and CD_3_OD. All of the chemical shifts were recorded and expressed in ppm units related to the TMS signal as an internal standard, and coupling constants (J) were recorded in Hz.

### 3.2. Sponge Collection 

*Callyspongia* sp. and *Spheciosponge vagabunda* were collected from the Red Sea (Ras Mohamed, Sinai; (GPS coordinates 27˚47.655 N; 34˚12.904 W) at a depth of 10 m in August 2006. The collected sponges, identified by R.W.M. van Soest (University of Amsterdam, Netherlands), were transferred to plastic bags containing sterile seawater and transported to the laboratory. Sponge biomass was cleaned with sterile seawater, cut into fragments of ca. 1 cm^3^, and then carefully homogenized with 10 volumes of seawater. The freshly prepared supernatant was diluted in 10-fold series (10^−1^, 10^−2^, 10^−3^) and subsequently plated out onto agar plates.

### 3.3. Actinomycetes Isolation 

Different media were used for isolation of actinomycetes M1 [39]—ISP2 medium [40], oligotrophic medium [41], and marine agar [42]. The isolation of slow-growing actinomycetes needed all these different media to be supplemented with nalidixic acid (25 µg/mL), nystatin (25 µg/mL), and 0.2 µm pore size filtered cycloheximide (100 µg/mL). Nystatin and cycloheximide prevent fungal growth, while nalidixic acid prevents many fast-growing Gram-negative bacteria [43]. All media consisted of Difco Bacto agar (18 g/L) and were prepared in 1 L artificial sterile sea water [44]. *Micromonospora* sp. UR56 and *Actinokinospora* sp. EG49 were cultivated on ISP2 medium. The inoculated agar plates were incubated at 30 °C for a long time, ranging from 6 to 8 weeks. Distinct colony morphotypes were selected and re-streaked many times until free of any contaminants. 

### 3.4. Molecular Identification 

16S rRNA gene amplification, cloning, and sequencing were performed using the universal primers 27F and 1492R according to Hentschel et al. [45]. Chimeric sequences were detected using the Pintail program [46]. The genus-level affiliation of the sequence was confirmed using the Ribosomal Database Project Classifier. The genus-level identification of all the sequences was performed using RDP Classifier (-g 16srrna, -f allrank) and confirmed with the SILVA Incremental Aligner (SINA) (search and classify option) [47]. An alignment was evaluated again using the SINA web aligner (variability profile: bacteria). Gap-only positions were removed with trimAL (-noallgaps). For phylogenetic tree construction, the best fitting model was first estimated using Model Generator. Visualization was performed using Interactive Tree of Life.

### 3.5. Microbial Fermentation and Extract Preparation

*Micromonospora* sp. UR 56 and *Actinokineospora* sp. EG49 were isolated from Red Sea sponges *Callyspongia* sp and *Spheciospongia vagabunda*, respectively. Each microbial strain was fermented in 10 Erlenmeyer flasks (2 L), each containing 1 L of ISP2 medium and incubated at 30 °C with shaking (150 rpm) for 14 days. For the co-fermentation experiment, 10 mL of 5 day old culture of *Micromonospora* sp. UR56 was transferred into 10 Erlenmeyer flasks (2 L), each containing 1 L of ISP2 medium inoculated with 10 mL of 5 day old culture of *Actinokineospora* sp. EG49. After fermentation of axenic cultures and co-culture, filtration was performed, and the supernatant was extracted with ethyl acetate (1.5 L) to give the ethyl acetate soluble fraction (700 mg).

### 3.6. LC-HR/MS Metabolomic Analysis

LC–HR–ESI–MS metabolomics analyses were performed as previously described by Abdelmohsen et al. [48]. Ethyl acetate soluble fraction 1 mg/mL in MeOH was uploaded and analyzed using an Accela HPLC (Thermo Fisher Scientific, Karlsruhe, Germany) combined with UV–visible detector and Exactive-Orbitrap mass spectrometer (Thermo Fisher Scientific, Karlsruhe, Germany) using an HPLC column (an ACE C18, 75 mm × 3.0 mm, 5 μm column (Hichrom Limited, Reading, UK).The gradient elution was carried out at 300 μL/min for 30 min using purified water (A) and acetonitrile (B) with 0.1% formic acid in each mobile phase. The gradient program started with 10% B, increased gradually to 100% B, and continued isocratic for 5 min before linearly decreasing back to 10% B for 1 min. The total analysis period for each fraction was 45 min. The injection volume was 10 μL and the column temperature was maintained at 20 °C. High-resolution mass spectrometry was performed in both negative and positive ionization modes with a spray voltage of 4.5 kV and capillary temperature of 320 °C. The mass range was maintained at 150–1500 *m*/*z*. All positive and negative ionization files used to cover the highest number of metabolites were subjected to data mining software MZmine 2.10 (Okinawa Institute of Science and Technology Graduate University, Japan) for deconvolution, peak picking, alignment, deisotoping, and molecular formula prediction. Dictionary of natural products (DNP), Marinlit, and METLIN databases were used for identification of all metabolites. 

### 3.7. Isolation and Purification of Induced Metabolites

The crude ethyl acetate (EtOAc) soluble fraction (700 mg, obtained from co-fermentation) was chromatographed on a Sephadex LH20 (32-64 µm, 100 × 25 mm) column using MeOH/H_2_O (80 : 20%) to afford five main fractions (Fr.1 - Fr.5). Fr.2 (200 mg) was chromatographed over a silica gel column (CC). Gradient elution was performed using a gradient mixture of DCM : EtOAc (100 : 0 to 0 : 100) followed by 100% MeOH to afford 13 subfractions. SubFr.5 (30 mg) was further chromatographed on a Sephadex LH20 using Hex: DCM (50:50) as mobile phase to yield compound **3** (15 mg, 2.14% crude weight). SubFr.7 was washed several times with DCM, resulting in crystallization and affording compound **9** (10 mg, 1.42% crude weight). Fr.3 (175 mg) was chromatographed over a silica gel column with *n*-hexane: EtOAc (100:0 to 0:100) to yield nine subfractions (Fr.3-1 to Fr.3-9). Fr.3-7 and Fr.3-8 (30 mg and 20 mg) were further chromatographed over Sephadex LH20 using Hex: CH_2_CL_2_ (50:50) as mobile phase to afford pure compounds **1** and **2** (12 mg and 15 mg, 1.71 and 2.14% crude weight), respectively. Fr.5 (75 mg) was further chromatographed on a Sephadex LH20 using MeOH as mobile phase to afford pure compound **10** (8 mg, 1.14% crude weight).

### 3.8. Assessment of Antibacterial Activity

The antibacterial activity of all isolated pure compounds (**1**–**3**, **9**, and **10**) was evaluated on Gram-positive pathogenic bacteria such as *Bacillus subtilis* (ATCC29212) and *Staphylococcus aureus* (ATCC9144 (and Gram-negative pathogenic bacteria such as *Escherichia coli*. (ATCC25922) and *Pseudomonas aeruginosa* (ATCC27853), as previously reported [49]. The tests were carried out in 96 well flat polystyrene plates. First, 10 μL of each isolated compound (15 µM) was transferred to 80 μL of lysogeny broth followed by the addition of 10 μL of bacterial suspension at log phase, and then all inoculated plates were incubated overnight at 37 °C. After incubation, the positive effect of the tested compounds was detected as clearance in the wells, and in compounds that showed no activity on the bacteria, the growth medium in the wells seemed opaque. The absorbance was observed after 20 h at OD600 in a Spectrostar Nano Microplate Reader (BMG LABTECH GmbH, Allmendgrun, Germany). The positive control was pathogenic bacteria plus distilled water, while the negative control was growth medium plus distilled water. 

### 3.9. Assessment of Antibiofilm Activity 

The biofilm-inhibitory activities of the isolated pure compounds (**1**–**3**, **9,** and **10**) were measured using 96 well flat polystyrene plates against four clinical microbes comprising Gram-positive (*Bacillus subtilis* and *Staphylococcus aureus*) and Gram-negative (*Escherichia coli* and *Pseudomonas aeruginosa*) pathogenic bacteria, according to Antunes, et al. [50] with some modifications. Briefly, each well was filled with 180 µL lysogeny broth (LB broth) and then inoculated with 10 µL of pathogenic bacteria followed by addition of 10 µL of sample (2 µM) along with control (without test sample). All inoculated plates were then incubated overnight at 37 °C and, after incubation, content in the wells was removed and wells were rinsed with 200 µL of phosphate-buffered saline (PBS) pH 7.2 to eliminate free-floating microbes and left to dry under sterilized laminar flow for 1 h. For staining, 200 µL/well of crystal violet (0.1%, w/v) was added, left for 1 h, and then excessive stain was removed and plates retained for drying. Further, dried plates were rinsed with 95% ethanol and optical density was measured at 570 nm using a Spectrostar Nano Microplate Reader (BMG LABTECH GmbH, Allmendgrun, Germany).

### 3.10. Assessment of Cytotoxic Activity 

#### MTT Assay

This assay was performed using different human cancer cell lines, including mammary gland breast cancer (MCF-7), colorectal carcinoma (HCT-116), human lung cancer cell line (WI38), and hepatocellular carcinoma (HePG-2). The cell lines were purchased from ATCC by (VACSERA), Cairo, Egypt. Doxorubicin was used as a positive control drug. The cell lines were used to detect the inhibitory effects of isolated compounds on cell growth. Cell viability was determined via colorimetric assay (MTT), which is based on the transformation of the yellow tetrazolium bromide to a purple formazan by mitochondrial succinate dehydrogenase. Cell lines were cultivated in RPMI-1640 medium supplemented with 10% fetal bovine serum, 100 units/mL penicillin, and 100 µg/mL streptomycin incubated at 37 °C under 5% CO_2_.The cell lines were plated in a 96 well microtiter plate at a concentration of 1.0 × 10^4^ cells/well and incubated at 37 °C under 5% CO_2_ for 2 days. Subsequently, the cells were mixed with different concentrations of the isolated compounds and incubated for a further 24 h, and then treated with 20 µL of MTT solution at a concentration of 5 mg/mL and incubated for 4 h. The purple formazan precipitates were dissolved in 100 µL of dimethyl sulfoxide (DMSO) and the optical density for each well was measured and recorded at an absorbance of 570 nm using a plate reader (EXL 800, Biotek^®^, Winooski, Vermont, USA). The relative cell viability as a percentage was calculated as A570 of treated samples/A570 of untreated sample × 100.

### 3.11. Enzyme Assays

DNA gyrase (type II topoisomerase) and topoisomerase IV (ParE) add negative supercoils into DNA using ATP hydrolysis as a source of energy. They are considered crucial bacterial enzymes that are absent in eukaryotes. DNA-gyrase-B- and ParE-inhibitory activities were evaluated using the Inspiralis assay kit (Inspiralis^®^, London, UK) on streptavidin-coated 96 well microtiter plates (Thermo Scientific, Hamburg, Germany), according to the manufacturer’s protocol [51]. The assay detects the ability of the isolated compounds to prevent the ATPase activity of both gyrase-B and ParE subunits. On the other hand, pyruvate kinase (PK) has been discovered to be an important hub protein in the interactome of MRSA [52]. The PK-inhibitory activity of the isolated compounds was assessed according to a previously reported method [53].

### 3.12. Docking Analysis

*Staphylococcus* Gyr-B and PK crystal structures with the of PDB codes 3g7b and 3T0T, respectively, were used. Docking experiments were performed using AutoDock Vina docking software [54]. Such docking engines deal with the receptor as a rigid structure and the ligand as a flexible structure during their calculations. The co-crystallized ligands were utilized to assign the binding sites. The ligand-to-binding-site shape-matching root mean square threshold was set to 2.0 Å. The interaction energies were determined using the Charmm force field (CFF) (v.1.02) with 10.0 Å as a non-bonded cutoff distance and distance-dependent dielectric. Subsequently, 5.0 Å was set as an energy grid extending from the binding site. The tested compounds were energy-minimized inside the selected binding pocket. The editing and visualization of the generated binding poses were performed using Pymol 2.3 software (Schrödinger, München, Germany).

### 3.13. Structuraal Elucidation of Isolated Compounds **1**–**3**, **9**, and **10.**

The structures of the known induced metabolites were confirmed by comparison of their spectroscopic (HRMS and ^1^H, ^13^C and 2D-NMR) data with the published literature data as **1**, dimethylphenazine-1,6-dicarboxylate [55]; **2**, phencomycin [55,56,57]; **3**, tubermycinB [57,58,59,60]; **9**, N-(2-hydroxyphenyl)-acetamide [24,56]; and **10**, *p*-anisamide [61].

## 4. Conclusions

Actinomycetes genomes, in particular the order Actinomycetales, consist of numerous biosynthetic gene clusters encoding for secondary metabolites that have not yet been detected under standard laboratory conditions, and that need to be cultivated using elicitation approaches such as co-cultivation. In the present work, two Red-Sea-sponge-associated actinomycetes, *Micromonospora* sp. UR56 and *Actinokineospora* sp. EG49, were subjected to co-cultivation. Metabolomic profiling of both axenic actinomycetes and their co-culture revealed that the latter was more diverse in its induced metabolites profile, particularly in terms of phenazine derivatives. After isolation of the major induced metabolites (**1**–**3**, **9**, and **10**), their possible antibacterial, antibiofilm, and cytotoxic activities were assessed. Phenazine derivatives **1**–**3** were significantly active as antibacterial and antibiofilm agents with mild cytotoxicity. Subsequently, they were tested against Gyr-B and PK as possible molecular targets. The presence of carboxylic acid or an ester moiety at C-1 and C-6 was found to be crucial for the activity spectra of these compounds, where the antibacterial and antibiofilm effects were flipped from Gram-positive to Gram-negative strains, and their enzyme-inhibitory activities were significantly decreased upon removal of either carboxylic acid or the ester moiety at C-6. These findings could represent a good starting point from which to develop further phenazine-based antibiotics. Additionally, they highlight microbial mixed fermentation as a simple and effective approach to produce new antimicrobial agents through the induction of otherwise cryptic bacterial biogenetic pathways.

## Figures and Tables

**Table 1:** Growth inhibition % of **1**–**3**, **9**, and **10** at 15 µM against different bacterial strains.

Tested Compounds	*Staphylococcus aureus*	*Bacillus subtilis*	*Escherichia coli*	*Pseudomonas aeruginosa*
**1**	57.19 ± 1.2	19.61 ± 1.5	9.93 ± 1.3	23.41 ± 1.7
**2**	68.87 ± 0.8	32.13 ± 1.3	11.15 ± 1.4	23.25 ± 2.2
**3**	NA	1.69 ± 1.2	24.34 ± 1.8	94.17 ± 2.8
**9**	1.1 ± 0.9	NA	NA	4.74 ± 1.6
**10**	53.17 ± 1.2	42.16 ± 1.9	19.27 ± 2.5	70.23 ± 1.1
**Gentamicin**	99.1 ± 0.7	97 ± 1.6	99 ± 0.6	99.7 ± 0.2

NA: no activity.

**Table 2:** Antibiofilm activity of **1**–**3**, **9**, and **10** at 2 µM against different bacterial strains.

Tested Compounds	*S. aureus*	*B. subtilis*	*E. coli*	*P. aeruginosa*
**1**	50.26 ± 0.4	12.10 ± 3.2	36.66 ± 2.9	18.36 ± 0.9
**2**	75.10 ± 2.4	18.65 ± 1.6	54.07 ± 2.5	22.28 ± 1.5
**3**	NA	NA	54.67 ± 1.4	93.98 ± 2.2
**9**	11.47 ± 2.9	4.91 ± 1.8	34.55 ± 2.6	7.39 ± 1.9
**10**	61.20 ± 3.7	20.29 ± 1.1	57.47 ± 3.1	73.52 ± 1.3

NA**:** no activity.

**Table 3:** Cytotoxic activity of isolated compounds **1**–**3**, **9**, and **10** at 2 µM.

Tested Compounds	WI38	HCT116	HePG-2	MCF7
**1**	63.18 ± 3.6	85.04 ± 3.9	92.06 ± 4.7	>100
**2**	76.30 ± 3.9	60.81 ± 3.5	76.11 ± 3.9	82.24 ± 4.4
**3**	51.22 ± 3.2	91.27 ± 4.6	>100	>100
**9**	36.47 ± 2.3	14.56 ± 1.2	10.16 ± 0.9	12.65 ± 1.1
**10**	>100	>100	>100	>100
Doxorubicin	6.72 ± 0.5	5.23 ± 0.3	4.50 ± 0.2	4.17±0.2

**Table 4:** Calculated interactions and affinities of compounds **1**–**3** with active-site residues of the *Staphylococcus* Gyr-B and PK.

Protein Target	Ligand	IC_50_ (µM) *	Binding Energy (kcal/mol)	Hydrogen Bonding Interactions	Hydrophobic Interactions
**Gyr-B**	**1**	19.18 ± 1.69	−7.6	ASN-54, GLU-58	ILE-51, VAL-79 PRO-87, ILE-86 ILE-102, ILE-103 ILE-175
	**2**	21.28 ± 2.36	−7.5	SER-55, ILE-51	ILE-51, VAL-79 PRO-87, ILE-86 ILE-102, ILE-103 ILE-175
	**3**	27.69 ± 1.08	−7.2	SER-55, ILE-51	ILE-51, VAL-79 PRO-87, ILE-86 ILE-102, ILE-103 ILE-175
	Co-crystallized ligand	0.091	-	ASP-81, ASN-54	ILE-51, VAL-79 PRO-87, ILE-86 ILE-102, ILE-103 ILE-175
**PK**	**1**	7.2 ± 0.07	−7.7	SER-362A, SER-362B THR-366A, THR-353B, ASN-369A	ALA-358B, ILE-361B, LEU-370A
	**2**	9.3 ± 0.03	−7.5	SER-362A, THR-366A, THR-353B, ASN-369A	ALA-358B, ILE-361B, LEU-370A
	**3**	22.5 ± 0.04	−6.5	ASN-369B, THR-353A	ALA-358A, ILE-361A
	Co-crystallized ligand	0.24	-	SER-362A, SER-362B, ASN-369B, HIS-365A	ALA-358A, ALA-358B ILE-361B, LEU-370A

* IC_50_ ± SD (µM).

## Supplementary Materials

The following are available online at https://www.mdpi.com/1660-3397/18/5/243/s1, Tables S1–S3: The dereplication results of the ethyl acetate fraction of micromonospora UR 56, Figures S1-S29: 1D and 2D NMR spectra of isolated compounds, Figures S30–S32: Dereplicated metabolites from metabolomic analysis.
