# Supplementary material for: Induction of Antibacterial Metabolites by Co-Cultivation of Two Red-Sea-Sponge-Associated Actinomycetes Micromonospora sp. UR56 and Actinokinespora sp. EG49
Source: Mar Drugs. 2020 May 5;18(5):243. doi: 10.3390/md18050243 (PMC7281614; doi:10.3390/md18050243)
Supplement: Supplementary file 1 [file marinedrugs-18-00243-s001.pdf]

Supplementary Material

Induction of Antibacterial Metabolites by Co-Cultivation of Two Red-Sea-Sponge-Associated Actinomycetes *Micromonospora* sp. UR56 and *Actinokinespora* sp. EG49

Mohamed S. Hifnawy[1§], Hossam M. Hassan[2§], Rabab Mohammed[2], Mohamed M. Fouda[3], Ahmed M. Sayed[3], Ahmed A. Hamed[4], Sameh F. AbouZid[2], Mostafa E. Rateb[5], Hani A. Alhadrami[6,7,*,] and Usama Ramadan Abdelmohsen[8,9*].

[1]*Department of Pharmacognosy, Faculty of Pharmacy, Cairo University, Cairo, Egypt 11787,*

[2]*Department of Pharmacognosy, Faculty of Pharmacy, Beni-Suef University, Beni-Suef, Egypt 62514,*

[3]*Department of Pharmacognosy, Faculty of Pharmacy, Nahda University, Beni-Suef, Egypt 62513,*

[4]*Microbial Chemistry Department, National Research Center, 33 El-Buhouth Street, 12622, Giza, Egypt,*

[5]*School of Computing, Engineering & Physical Sciences, University of the West of Scotland, Paisley PA1 2BE, UK,*

[6]*Department of Medical Laboratory Technology, Faculty of Applied Medical Sciences, King Abdulaziz University, Jeddah, 21589, Saudi Arabia,*

[7]*King Fahd Medical Research Centre, King Abdulaziz University, Jeddah, 21589, Saudi Arabia*

[8]*Department of Pharmacognosy, Faculty of Pharmacy, Minia University, 61519 Minia, Egypt,*

[9]*Department of Pharmacognosy, Faculty of Pharmacy, Deraya University, Universities Zone, P.O. Box 61111 New Minia City, 61519 Minia.*

*Correspondence: usama.ramadan@mu.edu.eg (URA) and hanialhadrami@kau.edu.sa (HAA).

§Equal contributions: Mohamed S. Hifnawy and Hossam M. Hassan as first authors

Table S1. The dereplication results of the ethyl acetate fraction of micromonospora UR 56.

Table S2. The dereplication results of the ethyl acetate fraction of Actinokinospora EG49.

Table S3. The dereplication results of the ethyl acetate fraction of co-culture.

Figure S1:^{1}H-NMR Spectrum of compound 1 (dimethyl phenazine-1,6-dicarboxylate)($CDCl_3$).

Figure S2: Expanded ^{1}H-NMR Spectrum of compound 1 **(**dimethyl phenazine-1,6-dicarboxylate)($CDCl_3$).

Figure S3: ^{1}H-^{1}HCOSY Spectrum of compound 1 (dimethyl phenazine-1,6-dicarboxylate)($CDCl_3$).

Figure S4: HSQC Spectrum of compound 1 (dimethyl phenazine-1,6-dicarboxylate)($CDCl_3$).

Figure S5: HMBC Spectrum of compound 1 (dimethyl phenazine-1,6-dicarboxylate ($CDCl_3$)

FigureS6: HRESIMS of compound 1 (dimethyl phenazine-1,6-dicarboxylate).

Figure S7:^{1}H-NMR Spectrum of compound 2 (phencomycin)(CDCl3).

Figure S8: Expanded ^{1}H-NMR Spectrum of compound 2 (phencomycin)($CDCl_3$).

Figure S9:^{13}C-NMR spectrum of compound 2 (phencomycin)($CDCl_3$).

Figure S10: ^{1}H-^{1}HCOSY Spectrum of compound 2 (phencomycin)($CDCl_3$).

Figure S11: HSQC Spectrum of compound 2 (phencomycin)($CDCl_3$).

FigureS12: HMBC Spectrum of compound 2 (phencomycin)($CDCl_3$).

FigureS13: HRESIMS of compound 2 (phencomycin).

Figure S14:^{1}H-NMR Spectrum of compound 3 (Phenazine -1-carboxylic acid)($CDCl_3$).

Figure S15: Expanded ^{1}H-NMR Spectrum of compound 3 (Phenazine -1-carboxylic acid($CDCl_3$).

Figure S16:^{13}C-NMR spectrum of compound 3 **(**Phenazine -1-carboxylic acid) ($CDCl_3$).

Figure S17: ^{1}H-^{1}HCOSY Spectrum of compound 3 **(**Phenazine -1-carboxylic acid) ($CDCl_3$).

Figure S18: HSQC Spectrum of compound 3 (Phenazine -1-carboxylic acid)($CDCl_3$).

FigureS19: HRESIMS of compound 3**(**Phenazine -1-carboxylic acid).

Figure S20:^{1}H-NMR Spectrum of compound 1 1 (N-(2-hydroxyphenyl)-acetamide)(MeOD).

Figure S21: Expanded ^{1}H-NMR Spectrum of compound 11 (N-(2-hydroxyphenyl)-acetamide) (MeOD).

Figure S22: Expanded ^{1}H-NMR Spectrum of compound 11 (N-(2-hydroxyphenyl)-acetamide) (MeOD).

Figure S23:^{13}C-NMR spectrum of compound 11 (N-(2-hydroxyphenyl)-acetamide)(MeOD).

Figure S24: ^{1}H-^{1}HCOSY Spectrum of compound 11 (N-(2-hydroxyphenyl)-acetamide)(MeOD).

Figure S25: HSQC Spectrum of compound 11(N-(2-hydroxyphenyl)-acetamide)(MeOD).

Figure S26: HMBC Spectrum of compound 11(N-(2-hydroxyphenyl)-acetamide)(MeOD).

FigureS27: HRESIMS of compound 11 (N-(2-hydroxyphenyl)-acetamide)(MeOD).

Figure S28:^{1}H-NMR Spectrum of compound 12 (P-anisamide)($CDCl_3$).

Figure S29: DEPTQ spectrum of compound 12(p-anisamide)($CDCL_3$) .

Figure S30:. Dereplicated metabolites from metabolomic analysis of. *Micromonospora* sp UR 56.

Figure S31: Dereplicated metabolites from metabolomic analysis of *Actinokineospora sp. EG49*.

Figure S32. Dereplicated metabolites from metabolomic analysis of ***co-culture*** *Micromonospora* sp. UR 56 and Actinokineospora *sp. EG49***.**

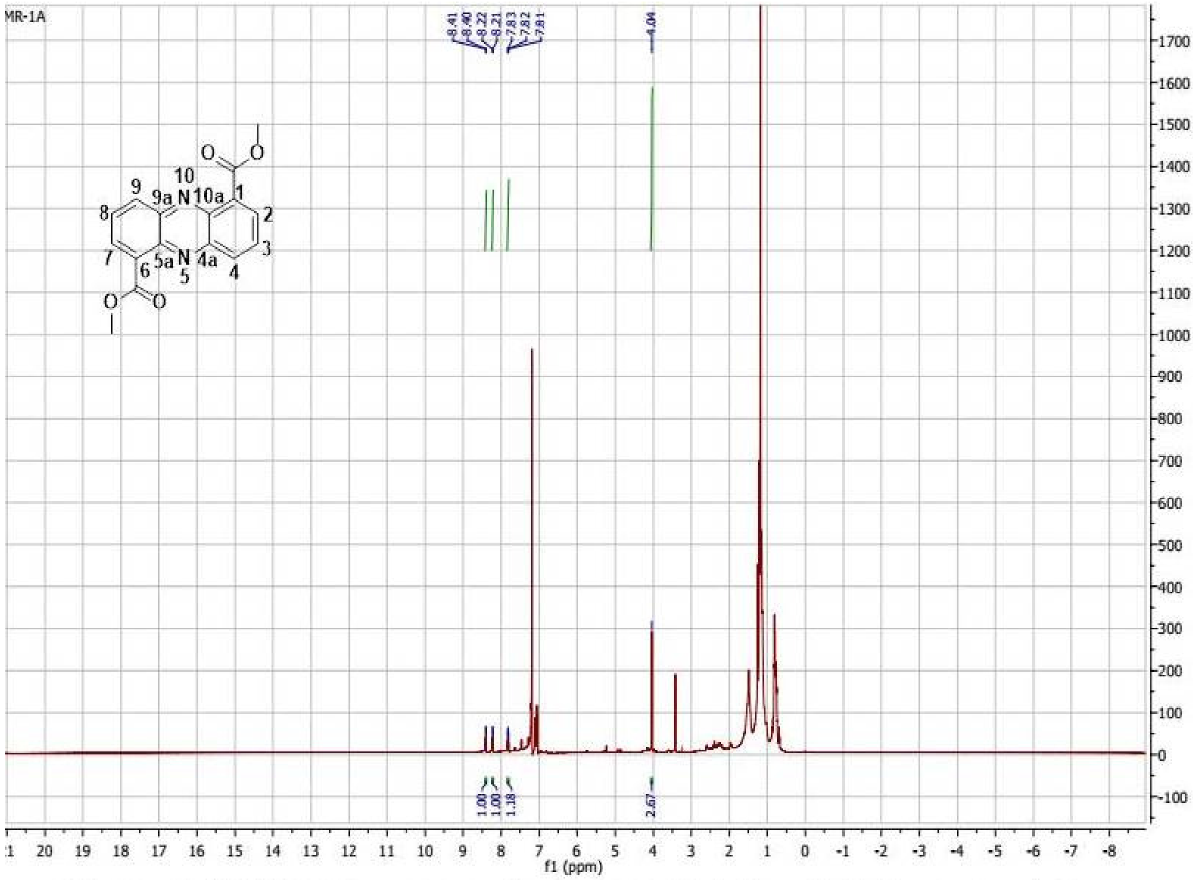

Figure S1:¹H-NMR Spectrum of compound 1 (dimethyl phenazine-1,6-dicarboxylate)($CDCl_3$).

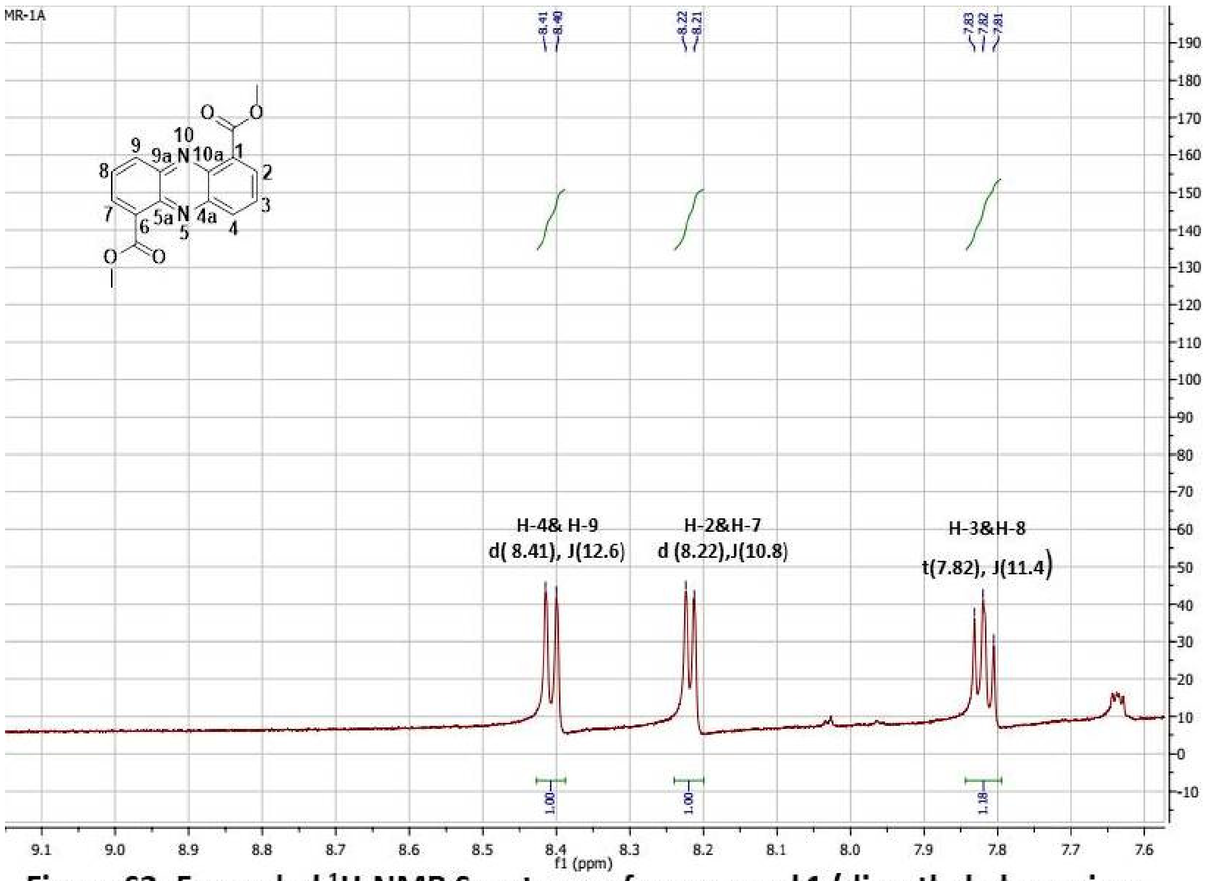

Figure S2: Expanded ^{1}H-NMR Spectrum of compound 1 (dimethyl phenazine-1,6-dicarboxylate)($CDCl_3$).

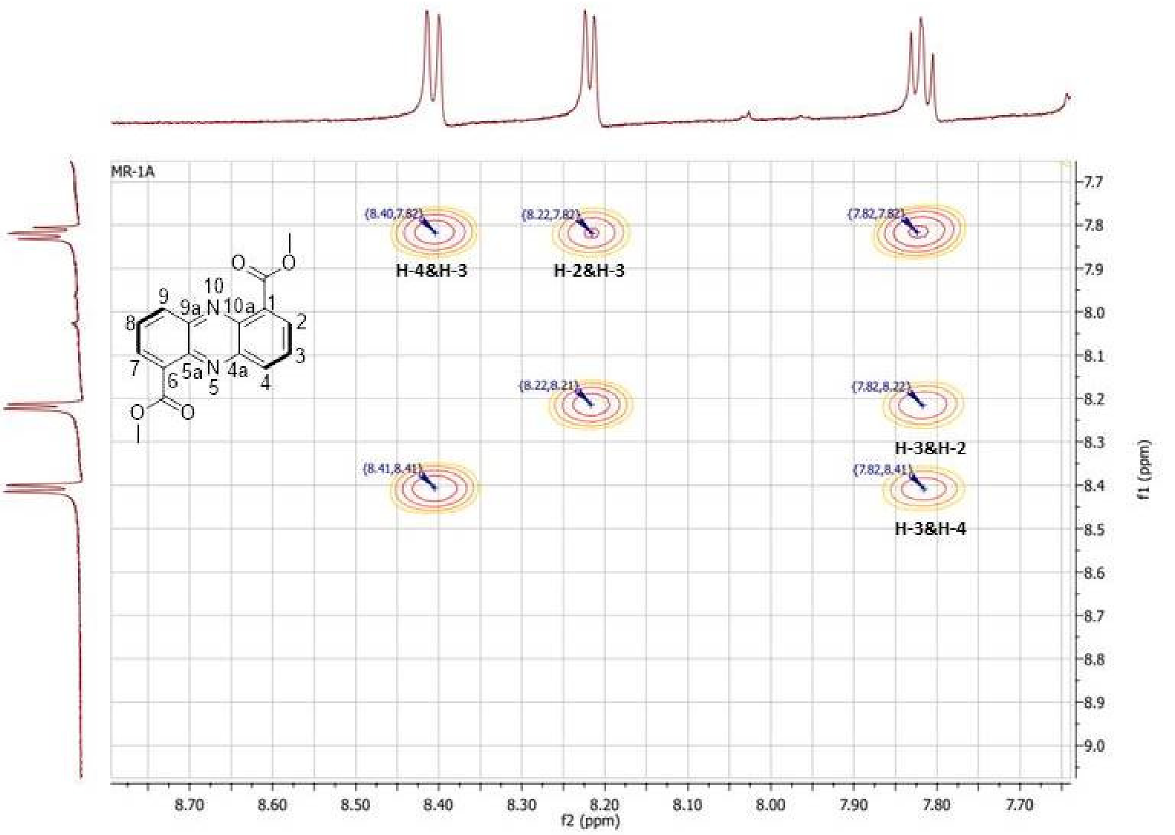

Figure S3: ^{1}H-^{1}HCOSY Spectrum of compound 1 (dimethyl phenazine-1,6-dicarboxylate)($CDCl_3$).

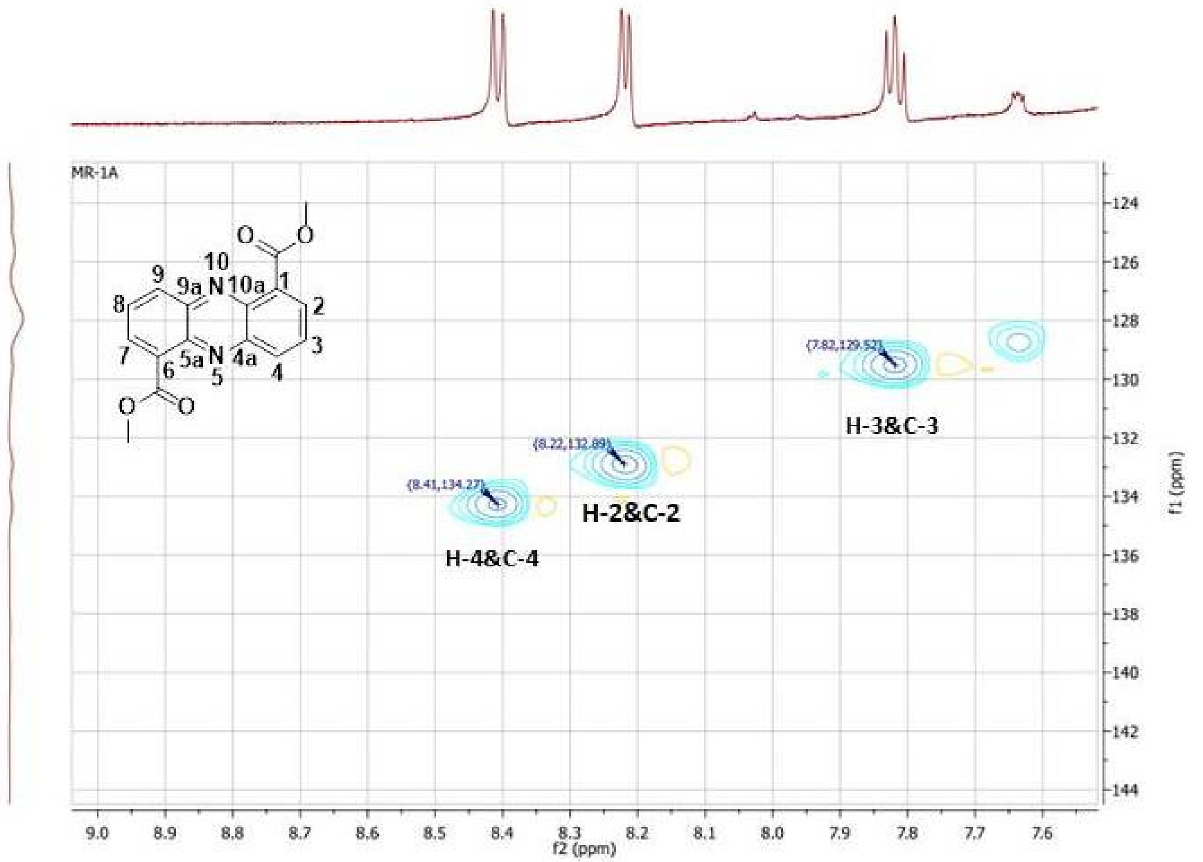

Figure S4: HSQC Spectrum of compound 1 (dimethyl phenazine-1,6-dicarboxylate)($CDCl_3$).

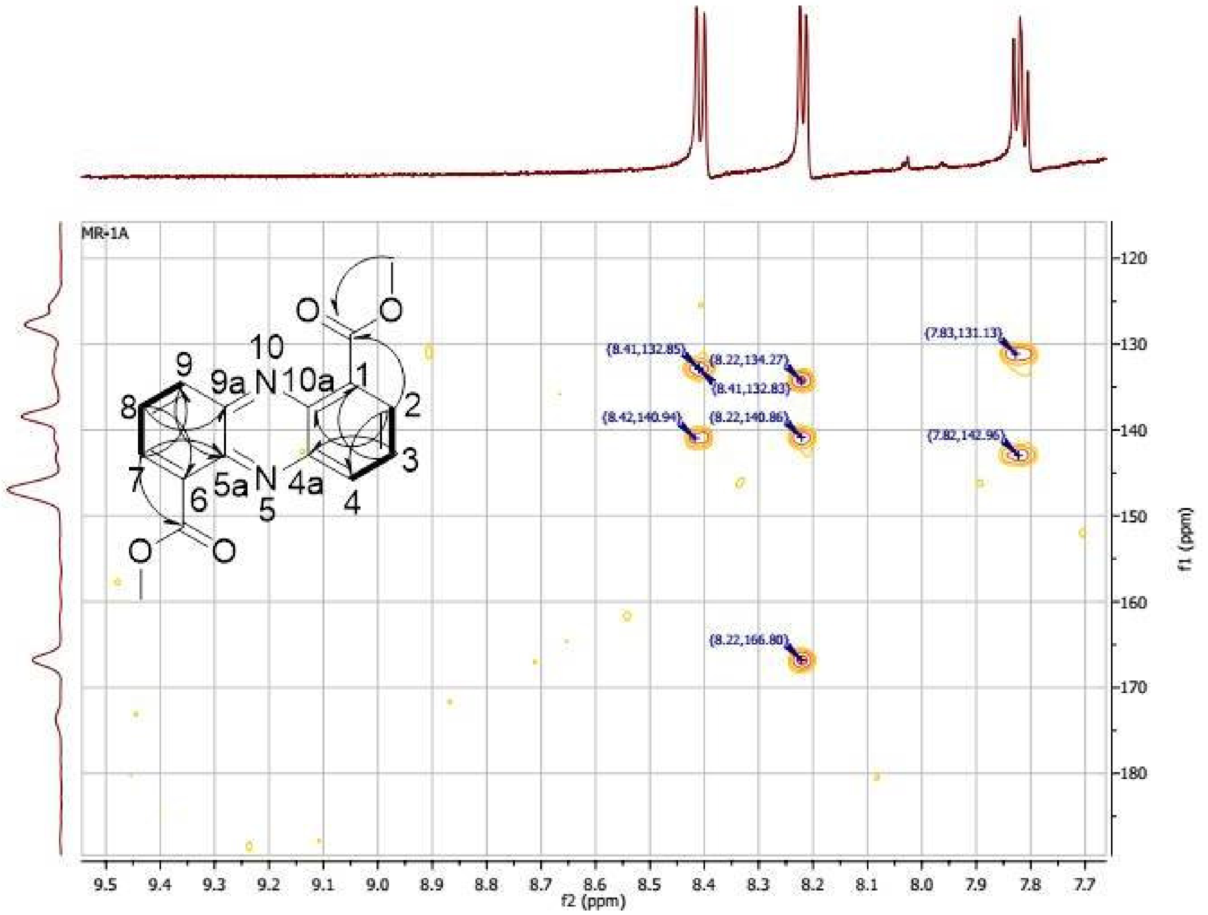

Figure S5: HMBC Spectrum of compound 1 (dimethyl phenazine-1,6-dicarboxylate ($CDCl_3$)

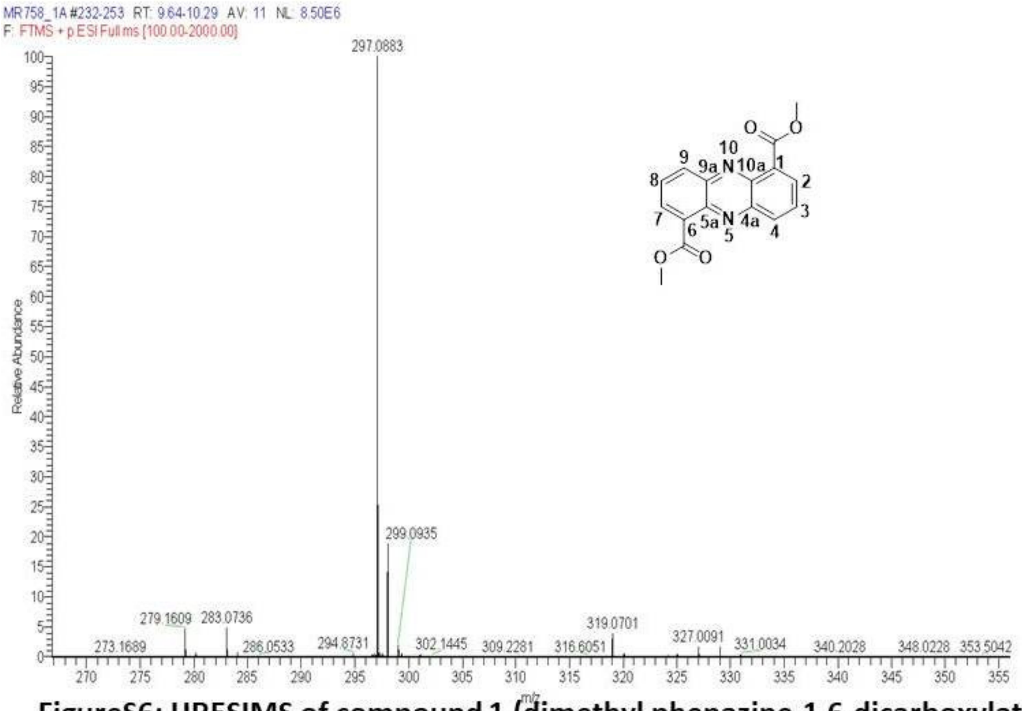

FigureS6: HRESIMS of compound 1 (dimethyl phenazine-1,6-dicarboxylate).

$C_{16}H_{13}N_2O_4$ (M+H)

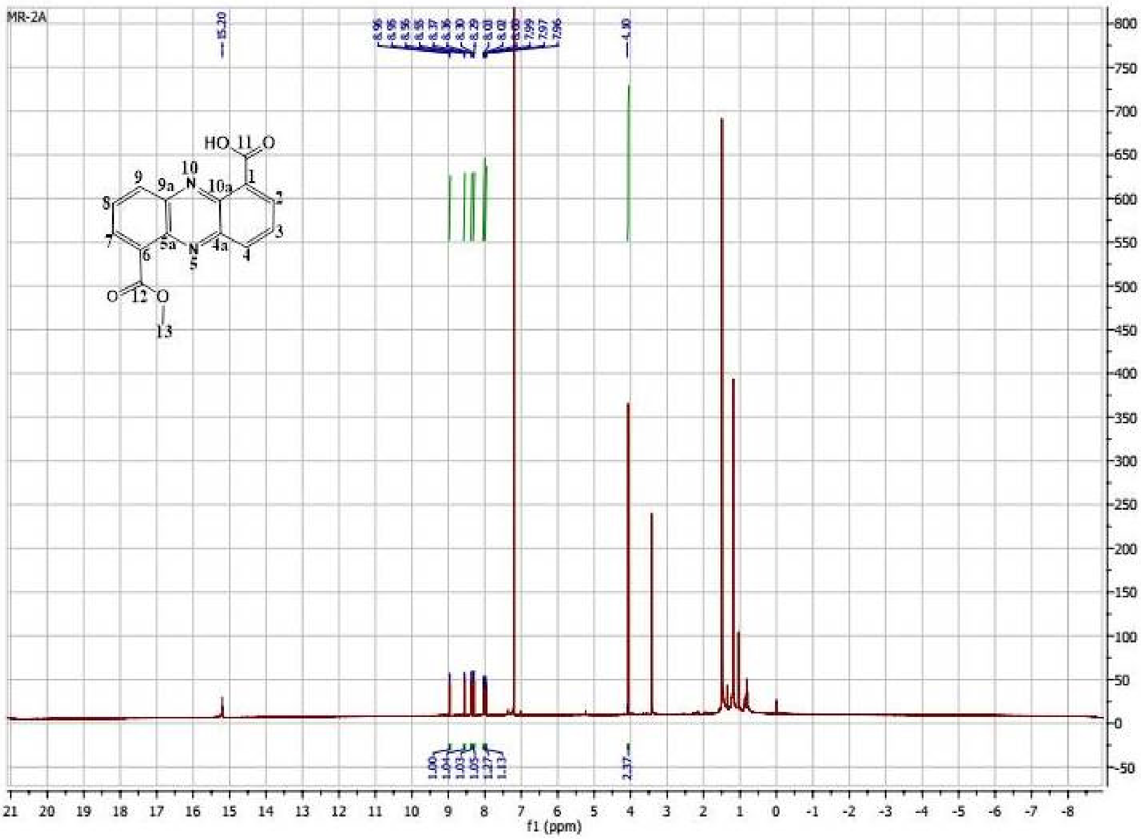

Figure S7:^{1}H-NMR Spectrum of compound 2 (phencomycin)(CDCl3).

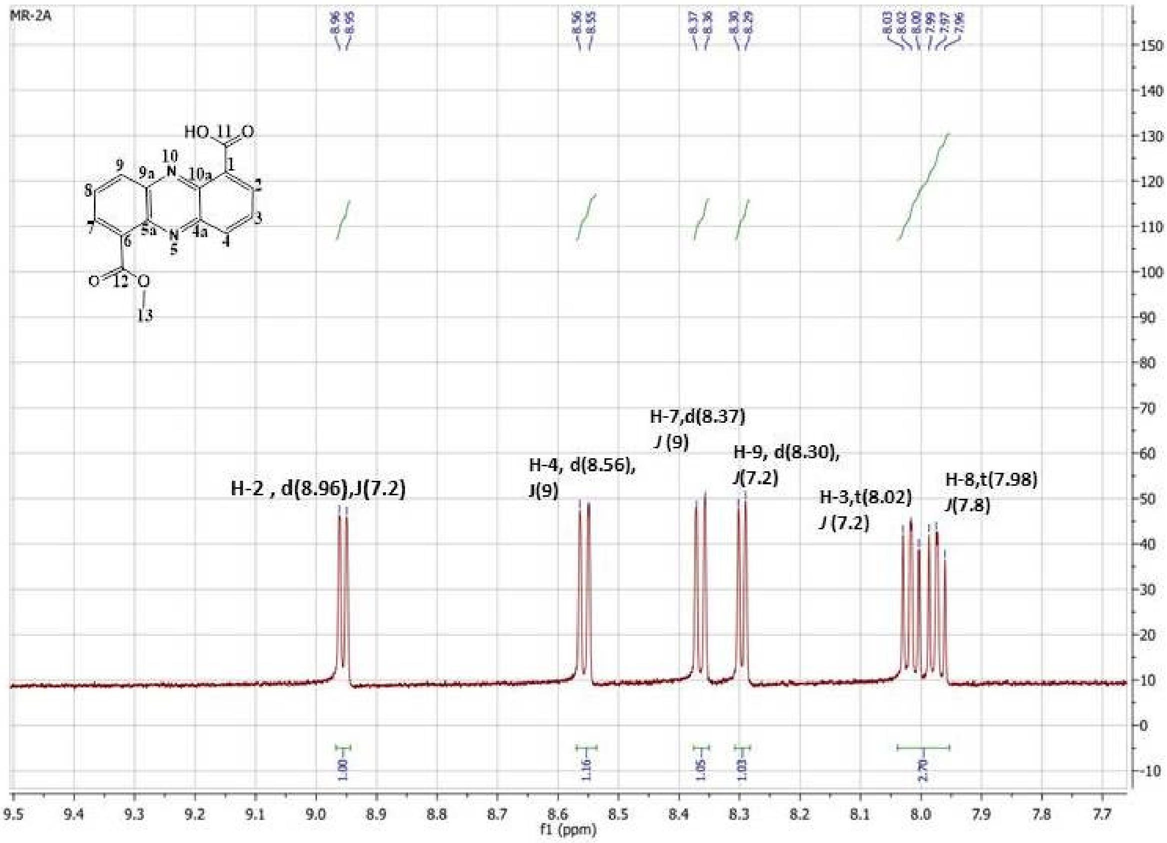

Figure S8: Expanded ^{1}H-NMR Spectrum of compound 2 (phencomycin)($CDCl_3$).

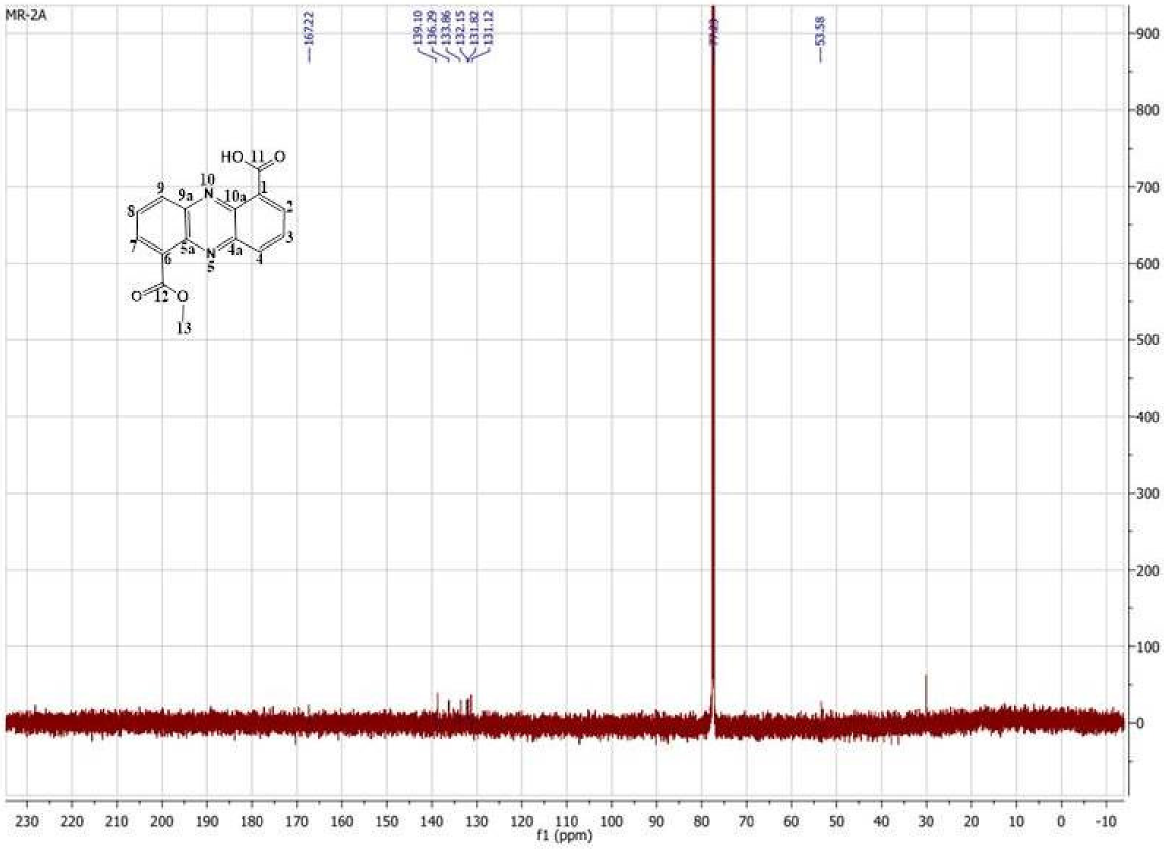

Figure S9:^{13}C-NMR spectrum of compound 2 (phencomycin)($CDCl_3$).

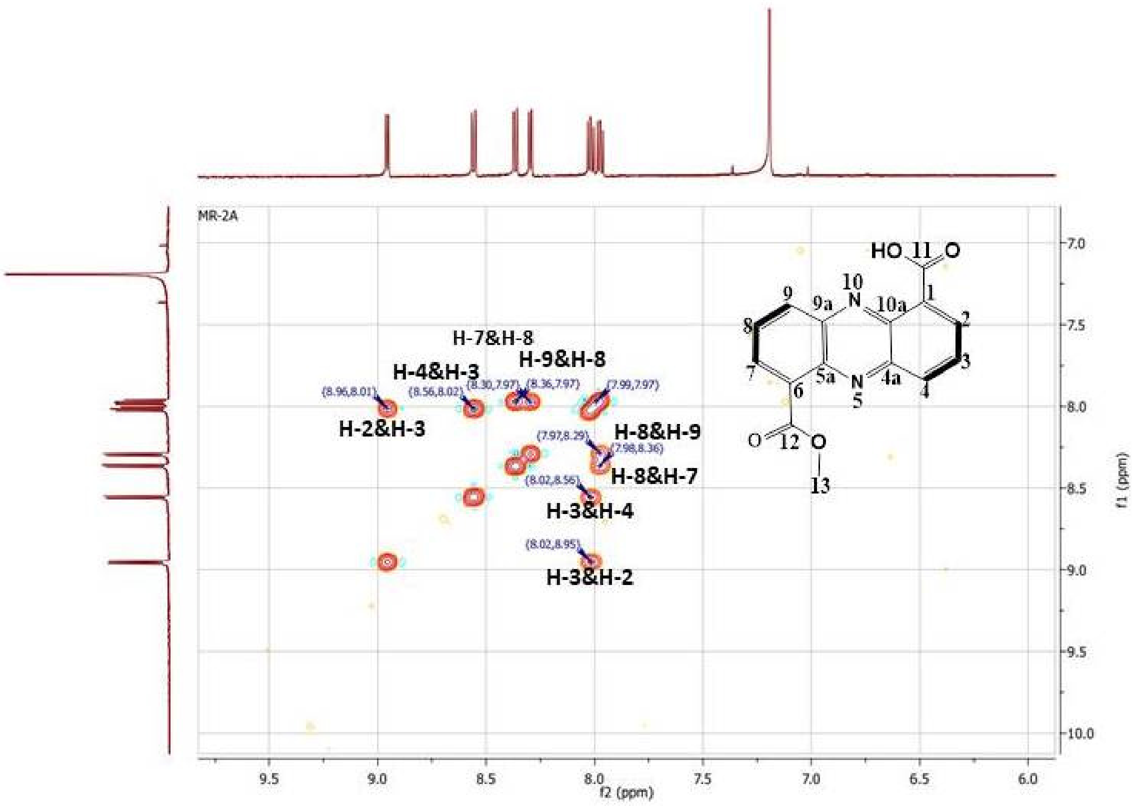

Figure S10: ^{1}H-^{1}HCOSY Spectrum of compound 2 (phencomycin)($CDCl_3$).

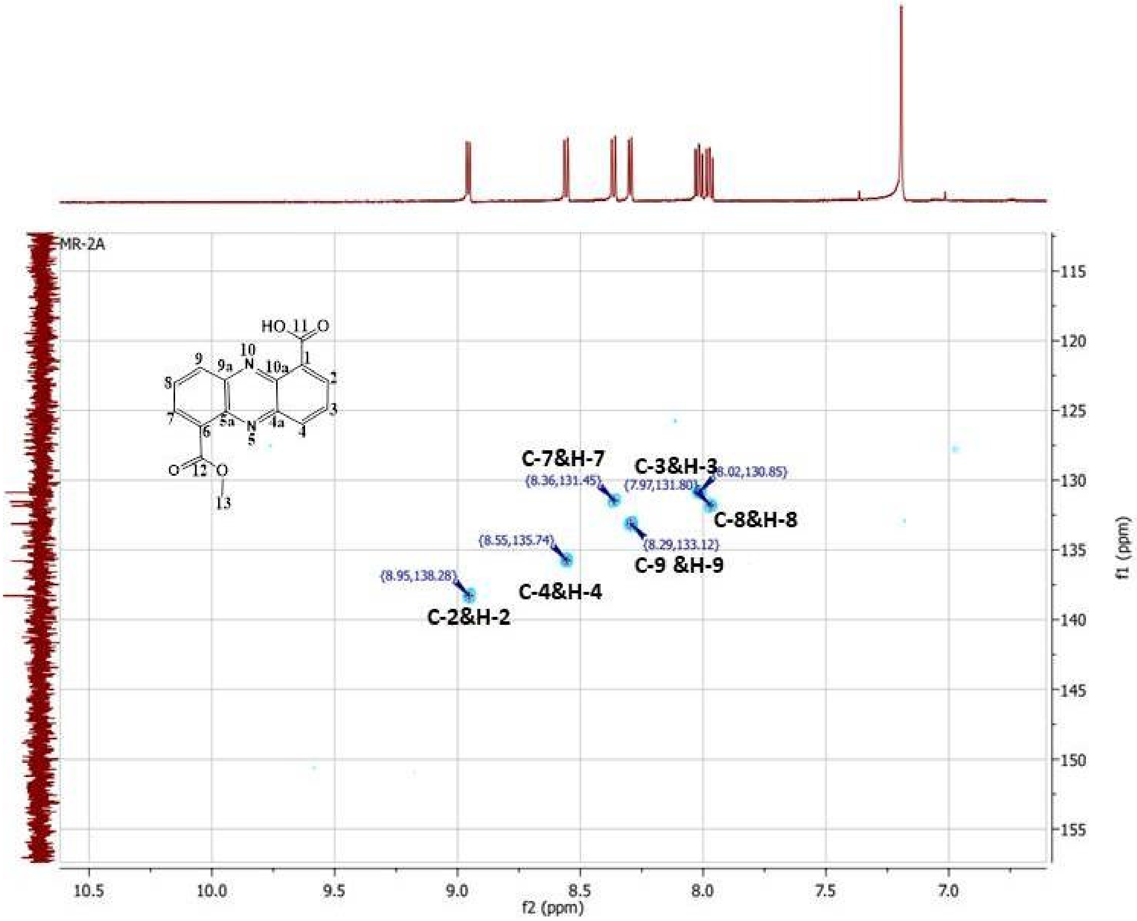

Figure S11: HSQC Spectrum of compound 2 (phencomycin)($CDCl_3$).

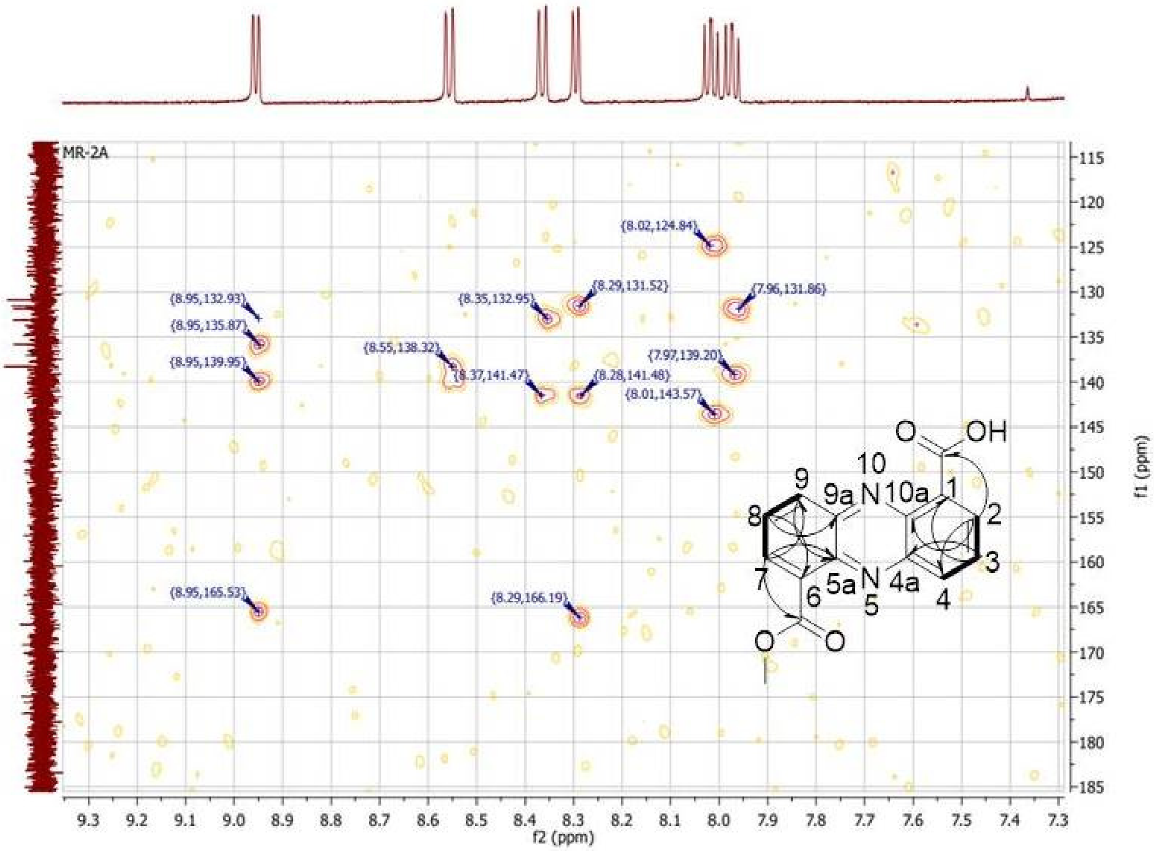

FigureS12: HMBC Spectrum of compound 2 (phencomycin)

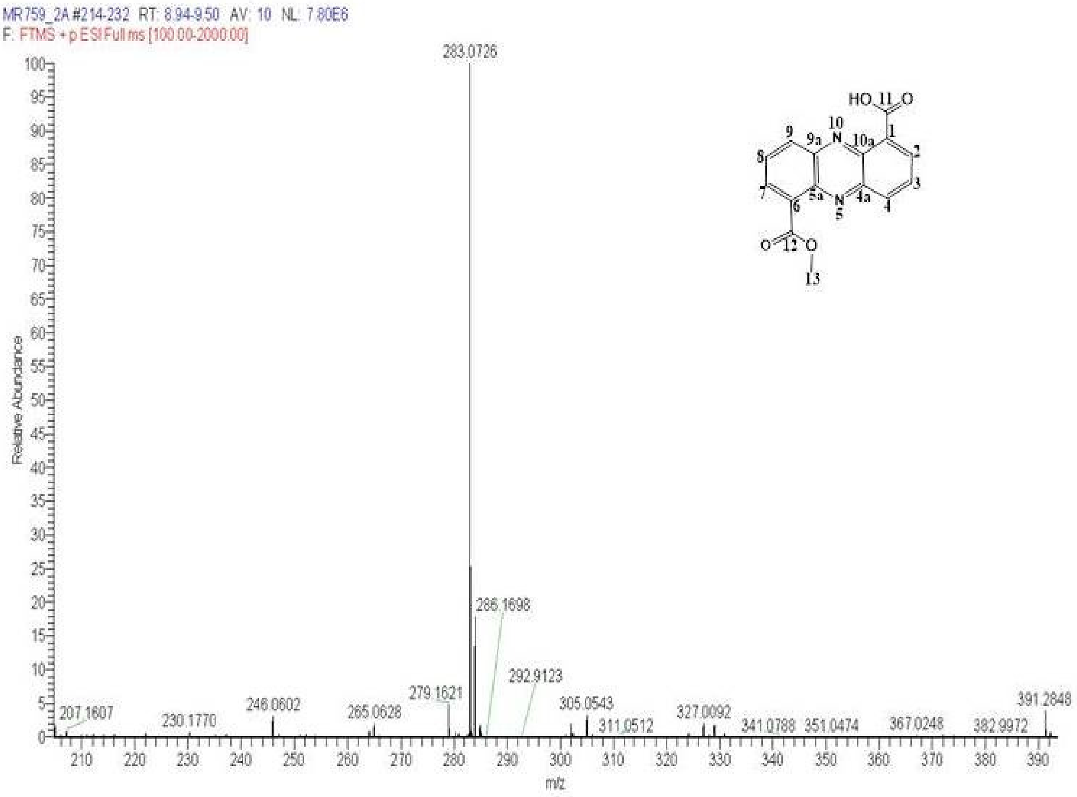

FigureS13: HRESIMS of compound 2 (phencomycin)

$C_{16}H_{14}N_2O_3$ (M+H)

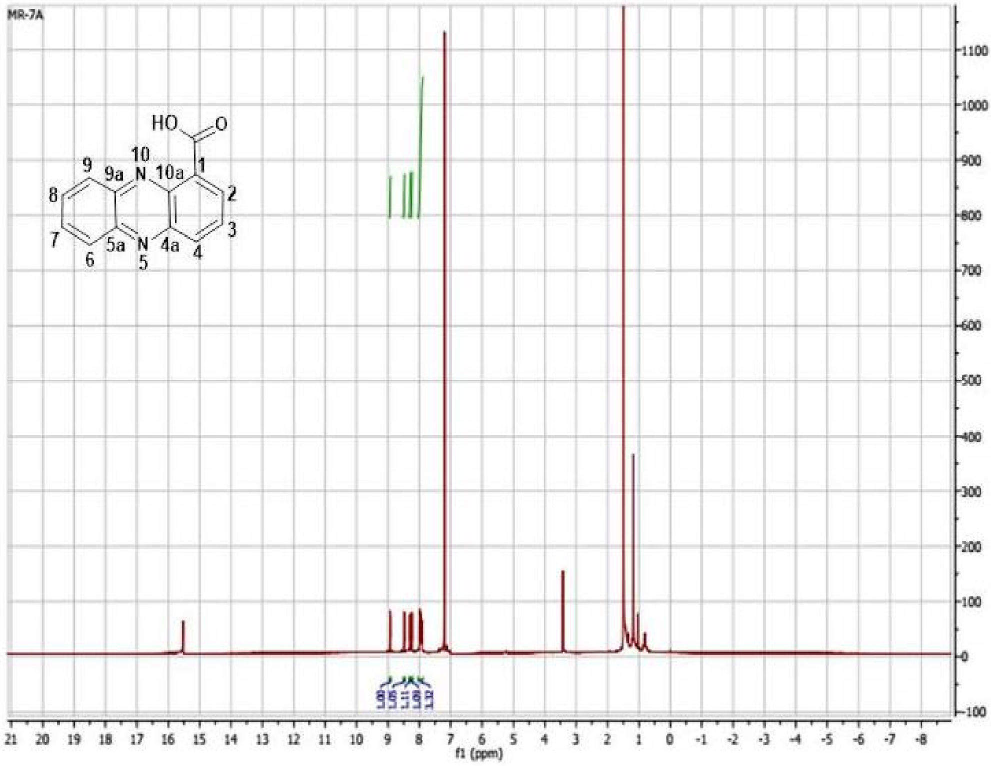

Figure S14:^{1}H-NMR Spectrum of compound 3 (Phenazine -1-carboxylic acid) (CDCl3).

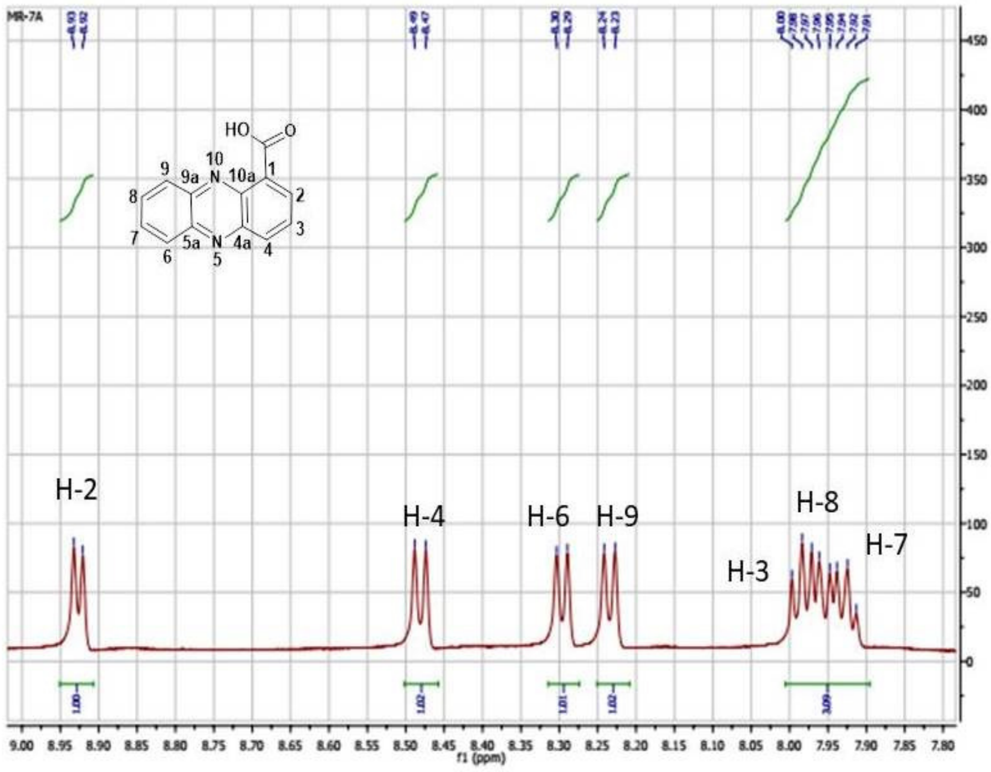

Figure S15: Expanded ^{1}H-NMR Spectrum of compound 3 (Phenazine -1-carboxylic acid) (CDCl3).

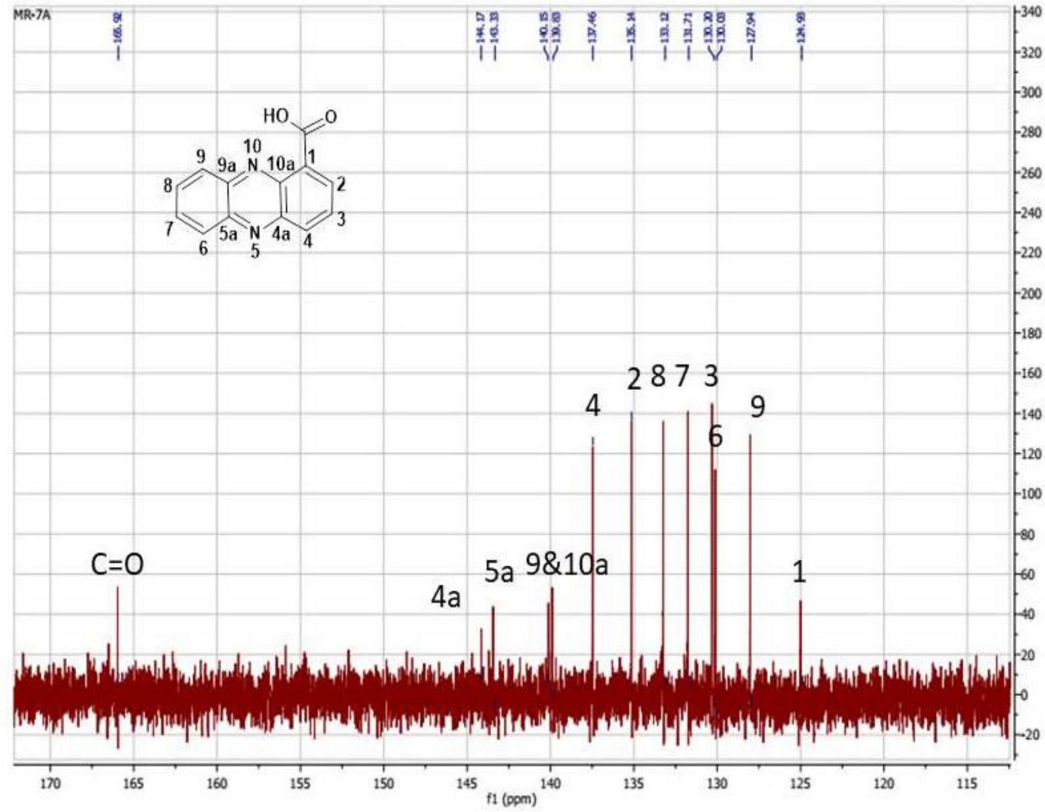

Figure S16:^{13}C-NMR spectrum of compound 3 (Phenazine -1-carboxylic acid) (CDCl3).

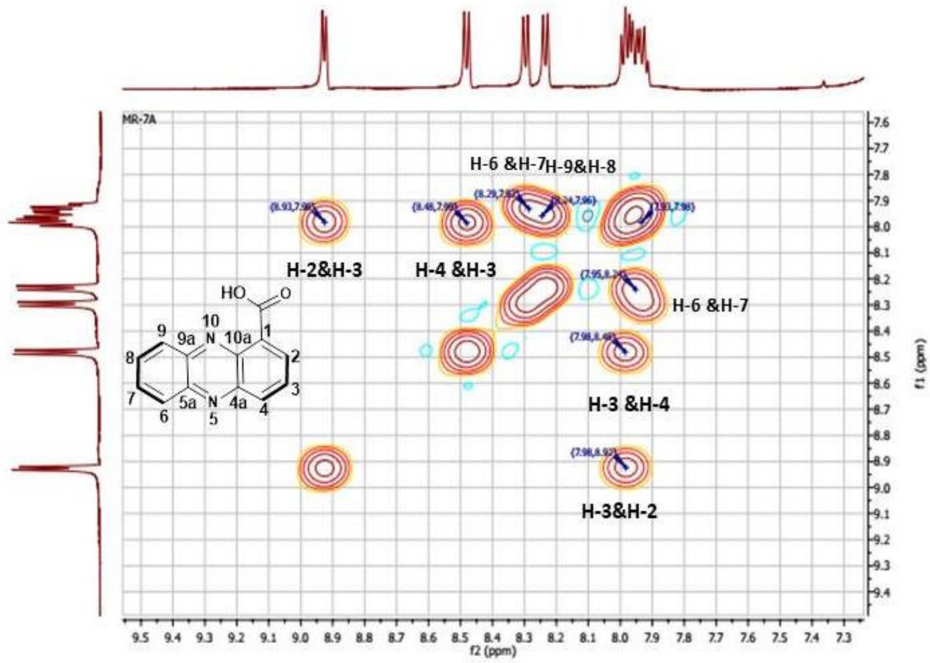

Figure S17: ^{1}H-^{1}HCOSY Spectrum of compound 3 (Phenazine -1-carboxylic acid) (CDCl3).

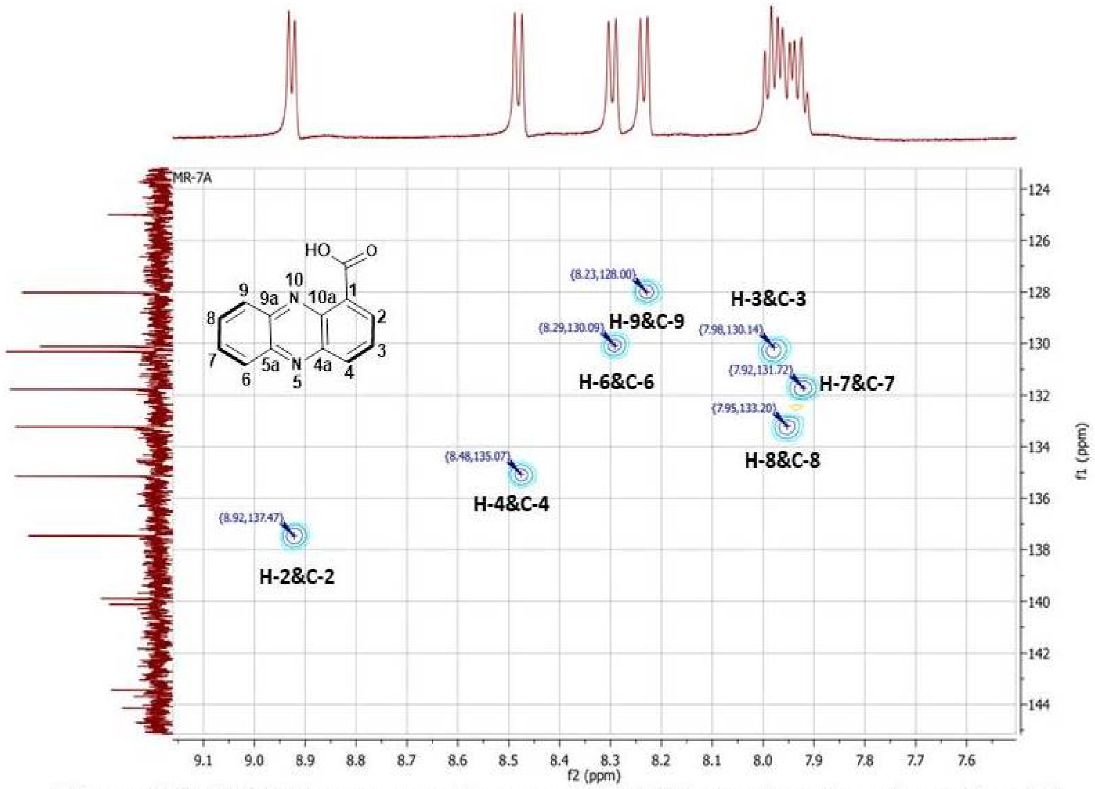

Figure S18: HSQC Spectrum of compound 3 (Phenazine -1-carboxylic acid) (CDCl3).

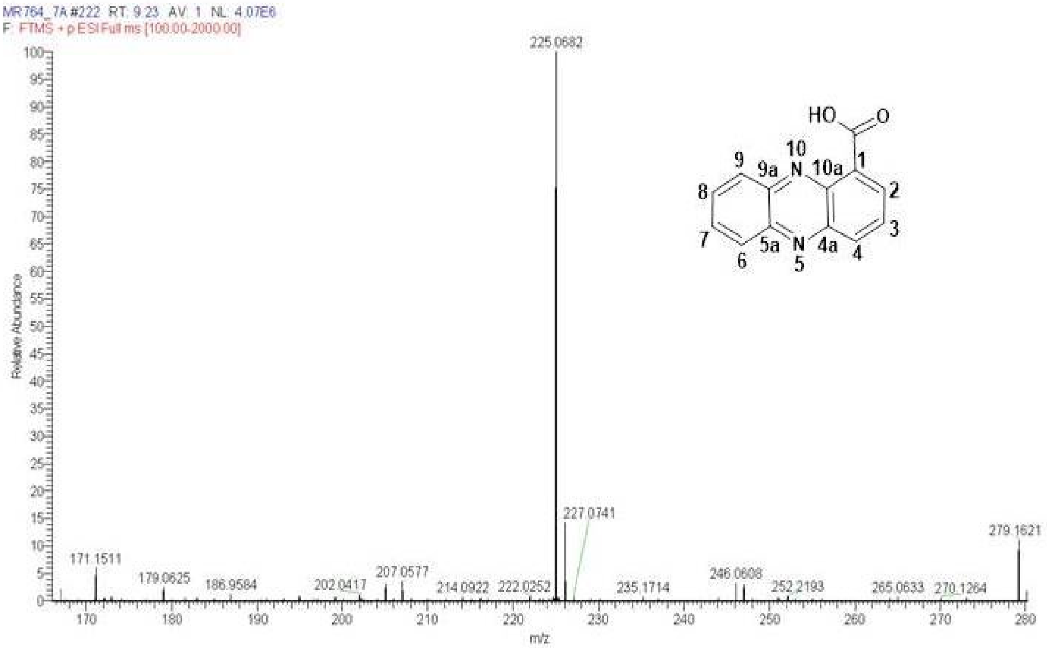

FigureS19: HRESIMS of compound 3(Phenazine -1-carboxylic acid).

$C_{13}H_{19}N_2O_2$ (M+H)

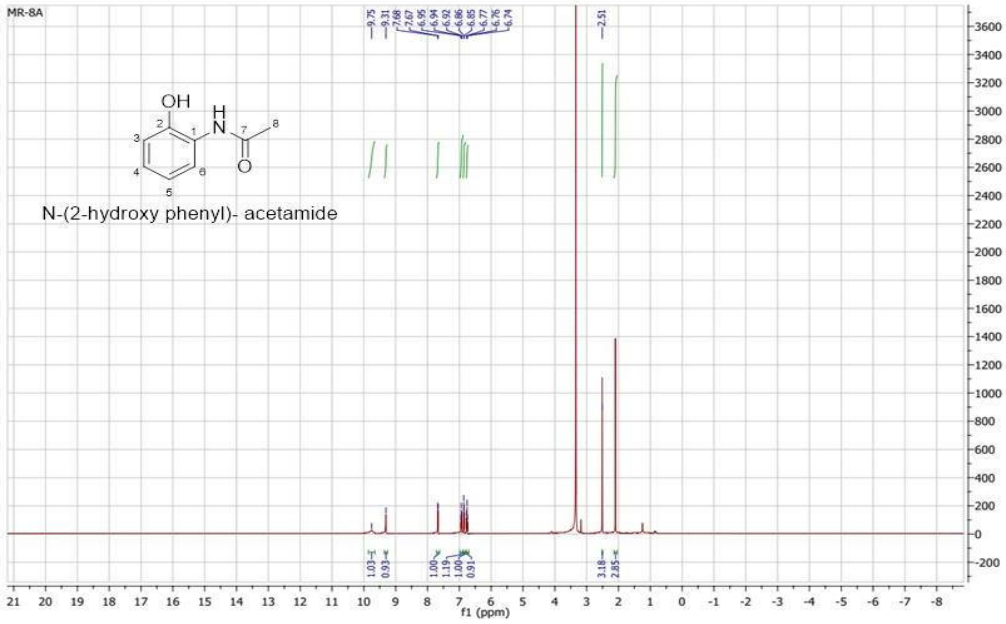

Figure S20:^{1}H-NMR Spectrum of compound 1 1 (N-(2-hydroxyphenyl)-acetamide) (MeOD).

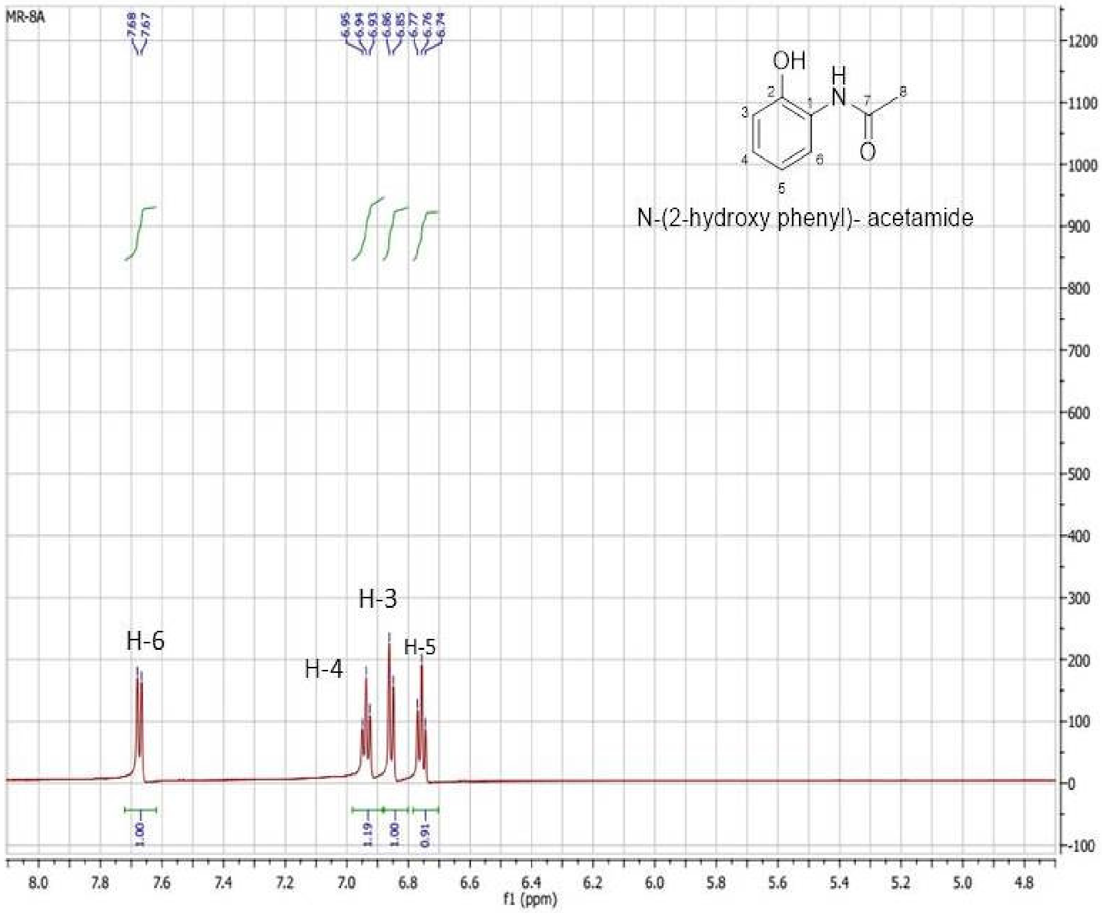

Figure S21: Expanded ^{1}H-NMR Spectrum of compound 11 (N-(2-hydroxyphenyl)-acetamide)(MeOD).

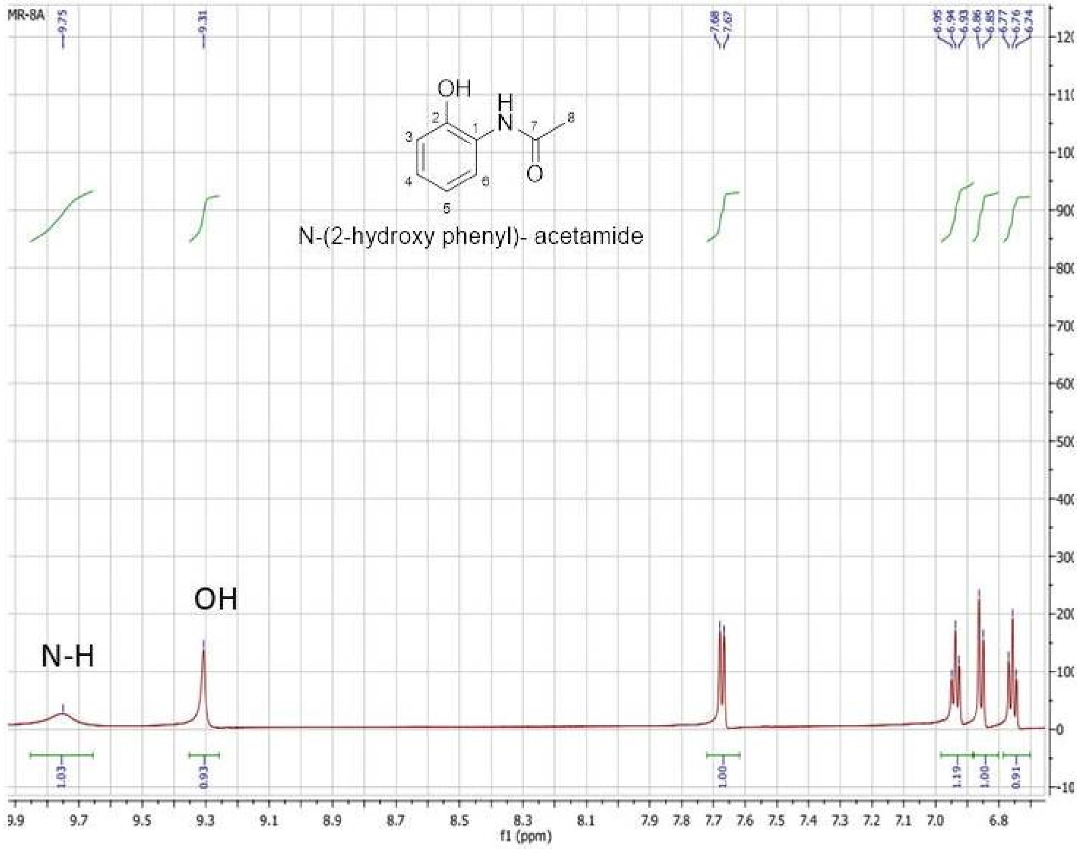

Figure S22: Expanded ^{1}H-NMR Spectrum of compound 11 (N-(2-hydroxyphenyl)-acetamide)(MeOD).

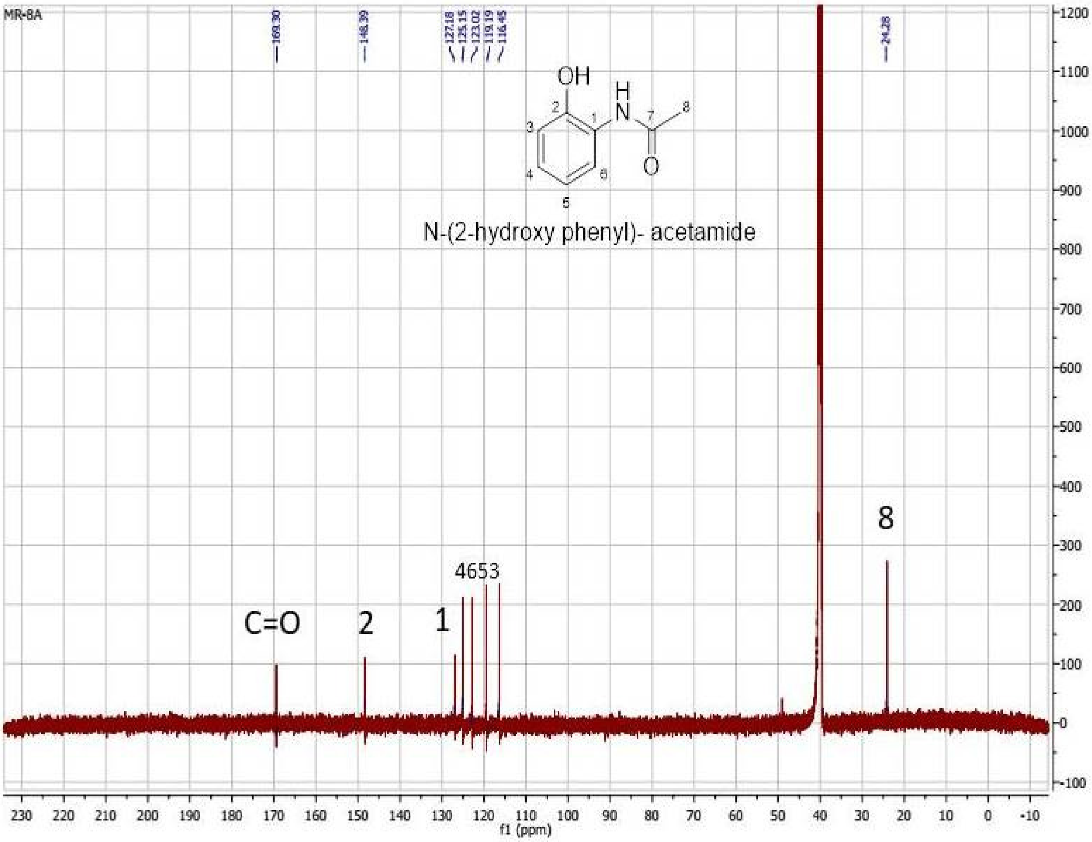

Figure S23:^{13}C-NMR spectrum of compound 11 (N-(2-hydroxyphenyl)-acetamide)(MeOD).

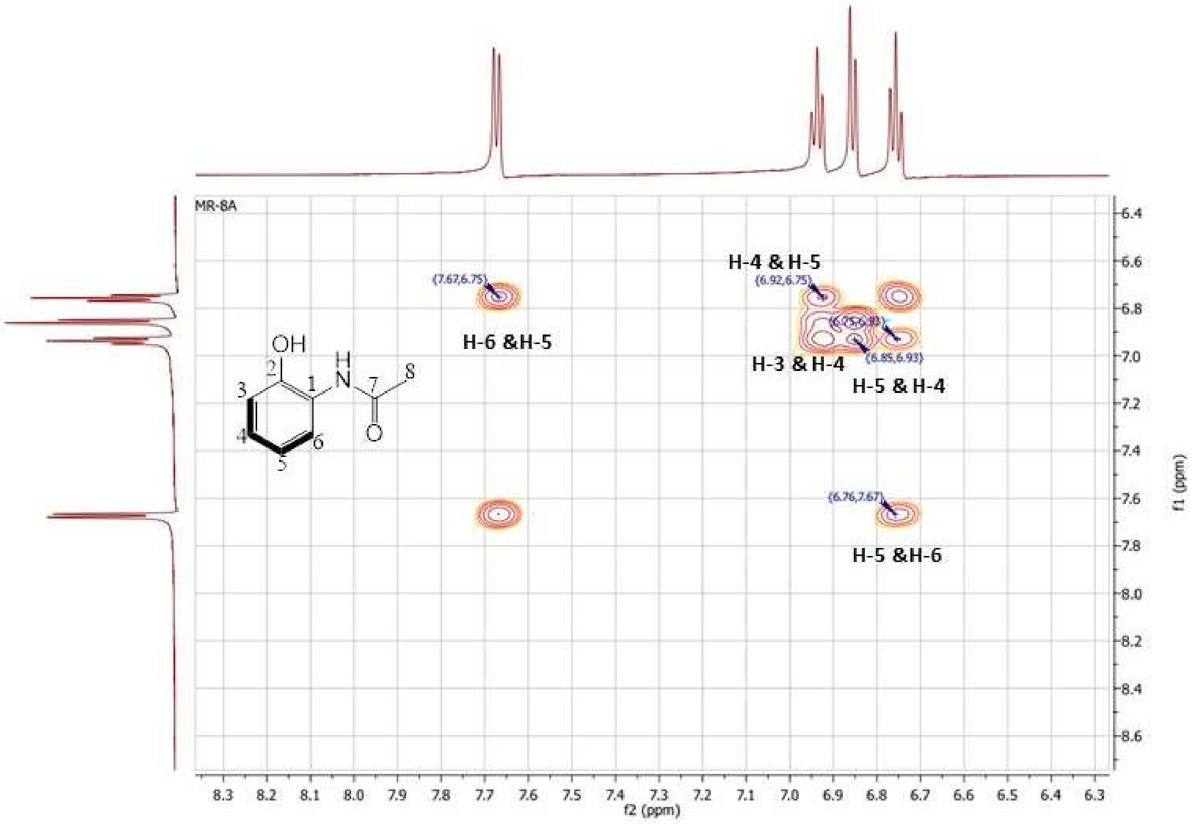

Figure S24: ^{1}H-^{1}HCOSY Spectrum of compound 11 (N-(2-hydroxyphenyl)-acetamide)(MeOD).

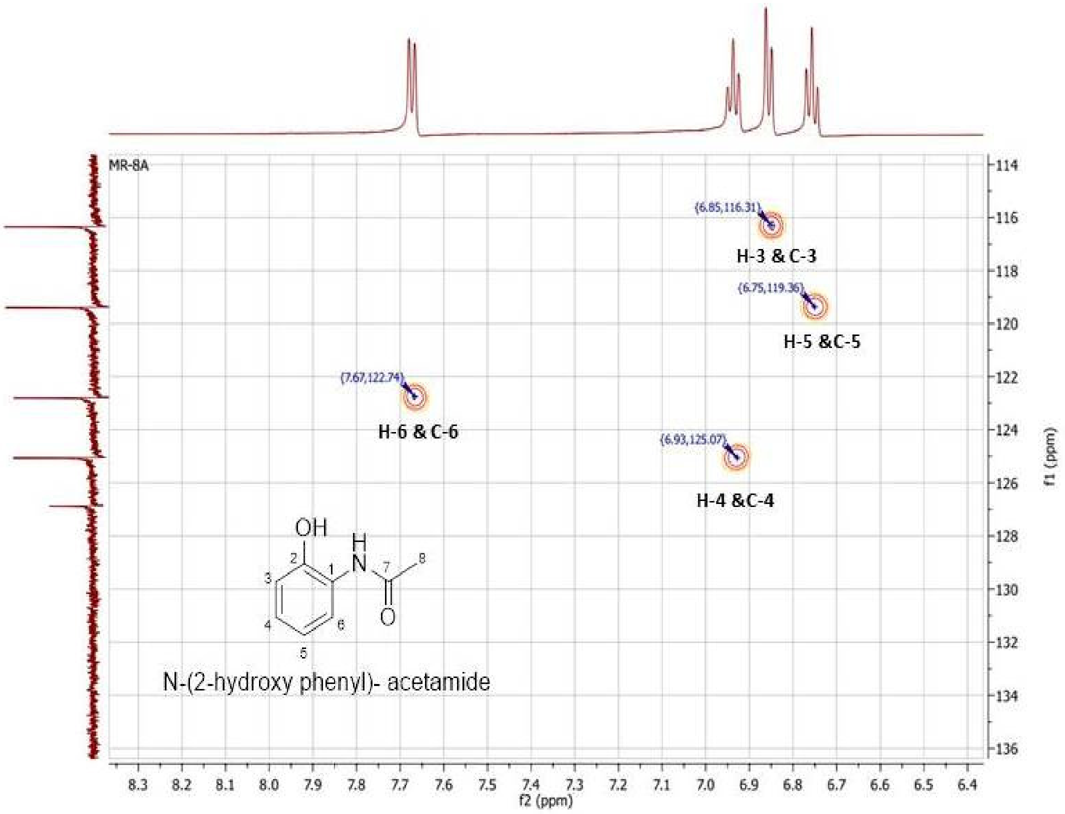

Figure S25: HSQC Spectrum of compound 11(N-(2 hydroxyphenyl)-acetamide)(MeOD).

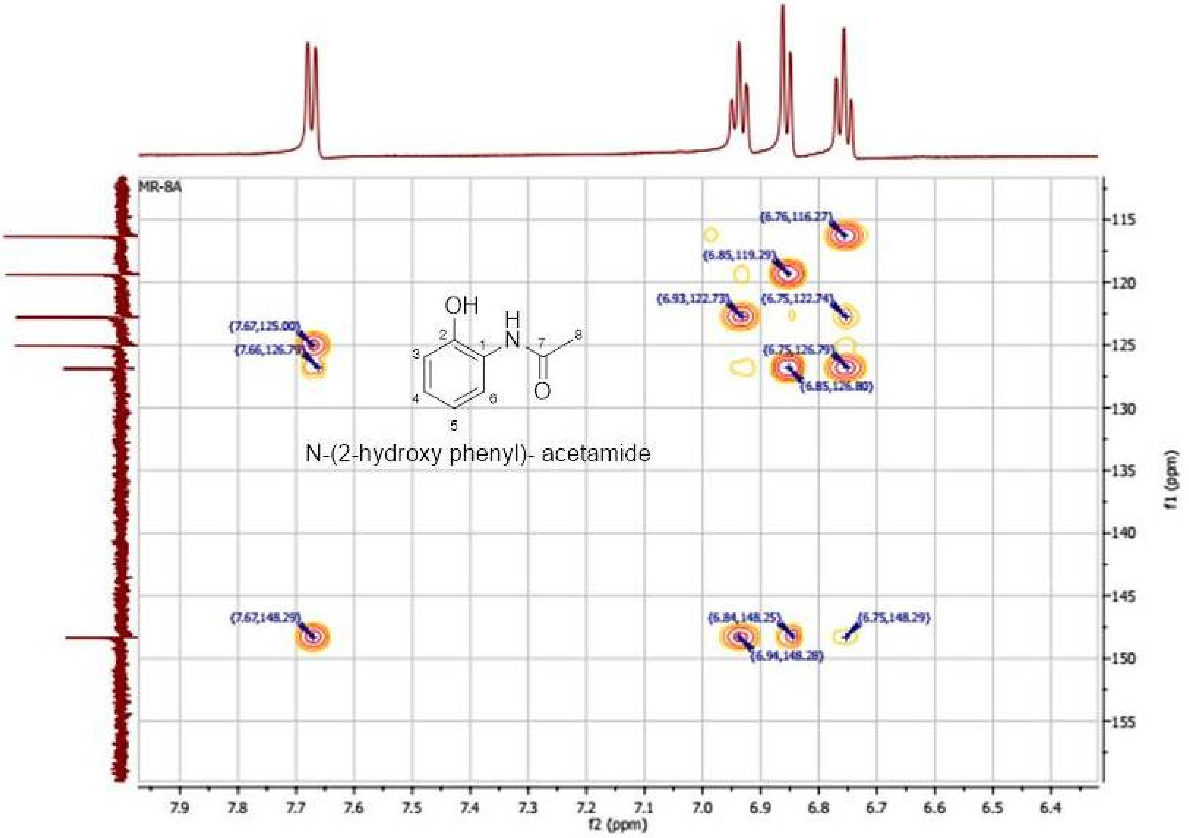

Figure S26: HMBC Spectrum of compound 11(N-(2-hydroxyphenyl)-acetamide)(MeOD).

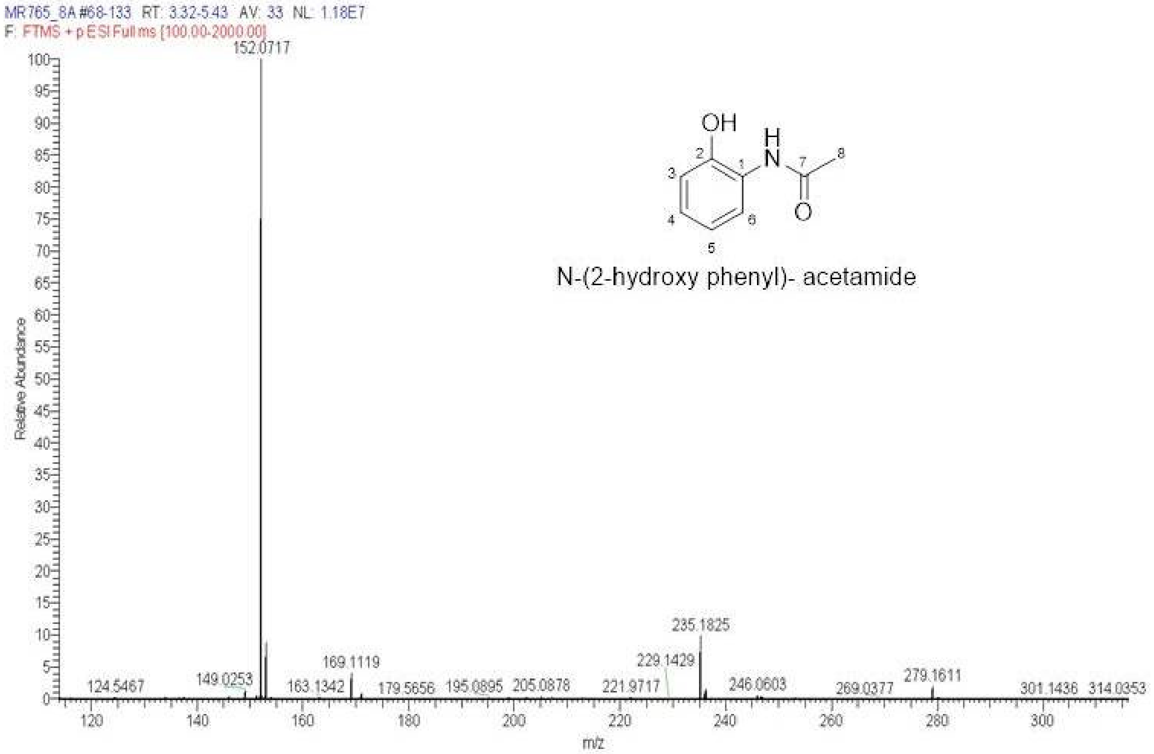

FigureS27: HRESIMS of compound 11 (N-(2-hydroxyphenyl)-acetamide)(MeOD). $C_8H_{10}O_2N$(M+H)

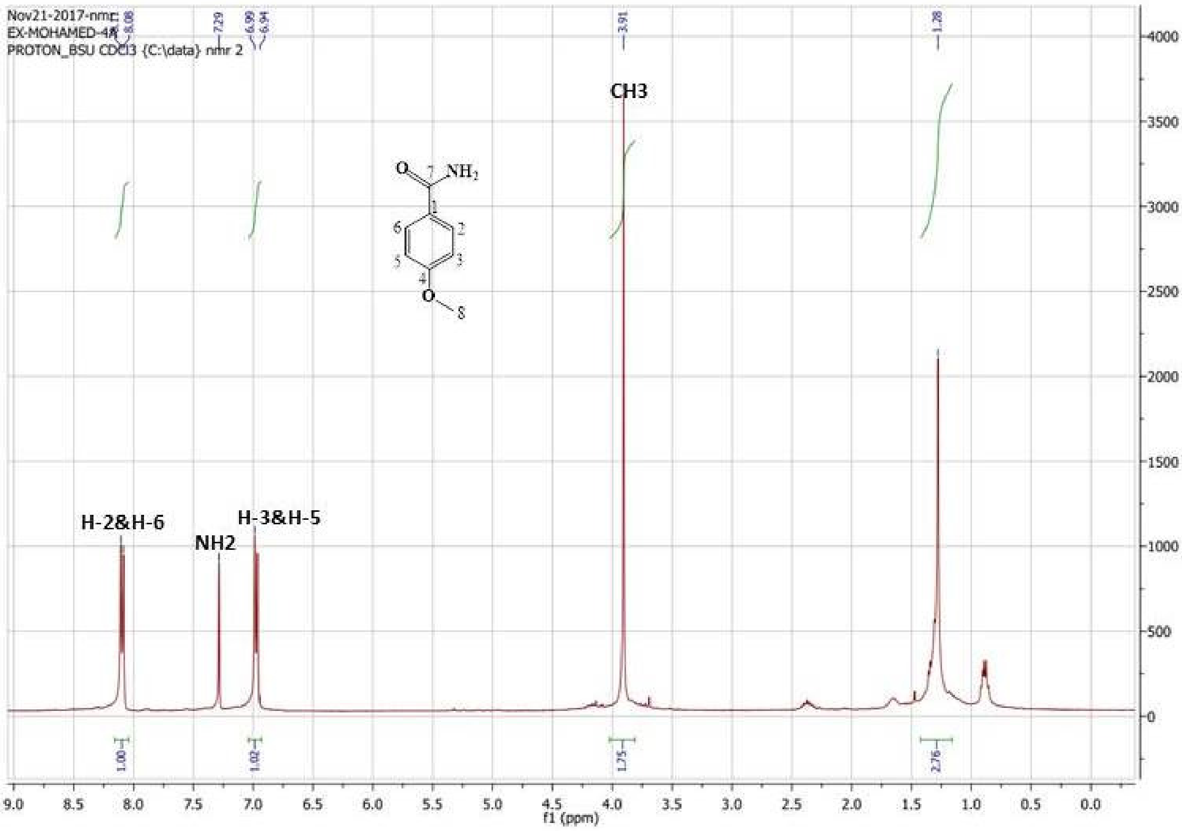

Figure S28:¹H-NMR Spectrum of compound 12 (P-anisamide)($CDCl_3$).

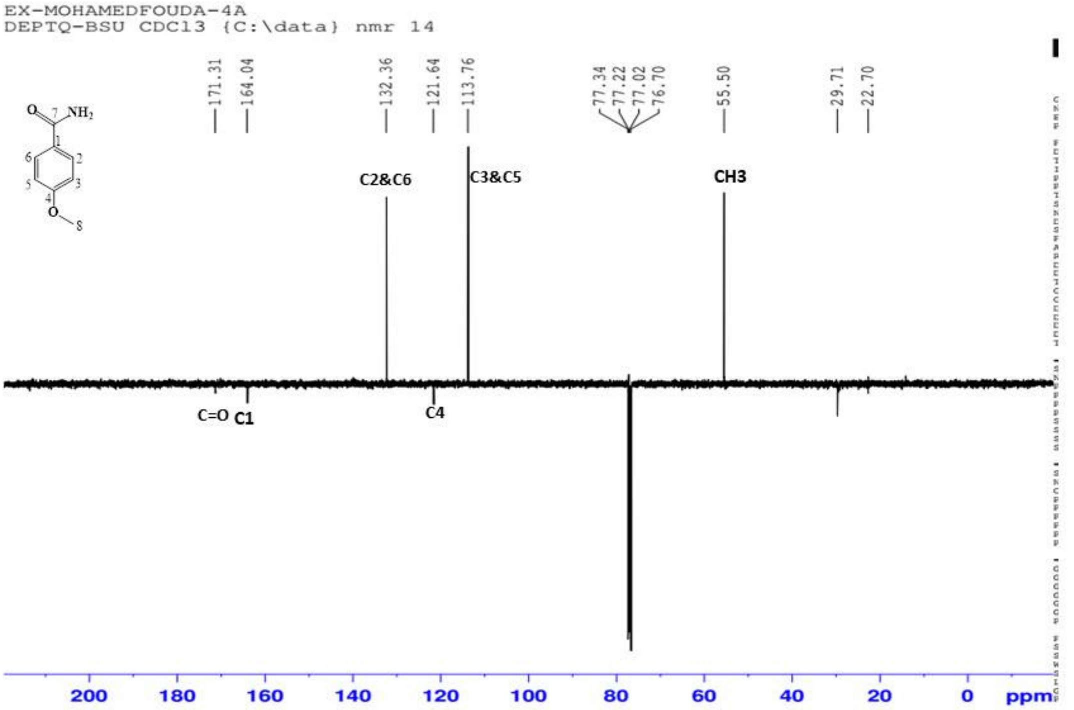

Figure S29: DEPTQ spectrum of compound 12(p-anisamide)($CDCL_3$) .

Table (S1). The dereplication results of the ethyl acetate fraction of micromonospora UR 56.

Polarity	Rt(min)	m/z	Formula	Name	Source	Class	Activity	Reference
**[M-H]$^-$**	4.48	253.0607	$C_{14}H_{10}N_2O_3$	9-Methoxy-1-phenazinecarboxylic acid. (1)	------------------	Phenazine	--------------	[1]
**[M-H]$^-$**	4.38	281.0557	$C_{15}H_{10}N_2O_4$	Griseoluteic acid (2)	Streptomyces y-9031725	Phenazine	Active against HeLa cells and Ehrlich carcinoma	[2]
**[M-H]$^-$**	4.44	341.0769	$C_{17}H_{14}N_2O_6$	Griseolutein A (3)	Streptomyces griseoluteus	Phenazine	Antibacterial activity against gram +ve and gram –ve bacteria	[3]
**[M-H]$^-$**	7.44	507.2489	C29H36N2O6	Aestivophoenin C (4)	Streptomyces purpeofuscus	Phenazine	Neuronal cell protecting agent, antioxidant	[4]
**[M+H]$^+$**	3.16	211.1447	$C_{11}H_{18}N_2O_2$	cyclo(D)-Pro-(D)-Leu (5)	Actinobacteria	diketopiperazine	Antifungal and antitumor	[5]
**[M-H]$^-$**	3.53	223.0600	$C_{11}H_{12}O_5$	Reticulol (6)	Streptomyces mobaraensis	benzopyrane	Inhibitor of cyclic nucleotide phosphodiesterase. Calmodulin antagonist	[6]
**[M-H]$^-$**	4.44	297.0506	$C_{15}H_{10}N_2O_5$	SE-212021 Umycin-A; SM-35 Carboxyexfoliazone (7)	Streptomyces sp. Streptomyces sp. DSM 3813	phenoxazine	Anthelmintic	[7]

**[M-H]-**	7.29	511.2799	$C_{29}H_{40}N_2O_6$	Dihydromaltophilin (8)	Streptomyces sp	Lactam	antifungal, angiogenesis inhibitor and antiulcer	[8]
**[M+H]+**	11.56	629.4235	$C_{34}H_{60}O_{10}$	X-14889-B; Ferensimycin-A; 5057-A (9)	Streptomyces sp. x-14889 (ATCC31679)	Poly ether	Active against Gram-positive bacteria. Effective in the treatment of coccidiosis of fowl	[9]
**[M-H]-**	5.82	670.3025	$C_{39}H_{45}NO_9$	Naphthomycin C (10)	Streptomyces collinus Tue 2357	Ansamycin	Antibacterial and antifungal	[10]
**[M-H]-**	12.91	1042.6429	$C_{53}H_{93}N_3O_{17}$	Neocopiamycin A (12)	Streptomyces hygroscopicus var. crystallogenes ifm 1136 (ATCC 19040)	Lactone	Antifungal	[11]
**[M+H]+**	11.62	598.1100	$C_{32}H_{22}O_{12}$	Crisamicin A (13)	Micromonospo ra m. purpureochro mogenes celinoensis	Quinone	Active against Gram-positive bacteria. Immunomodula tor	[12]

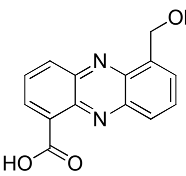

$C_{14}H_{10}N_2O_3$
Exact mass:254.0691
1-hydroxymethyl-6-carboxy phenazine
(1)

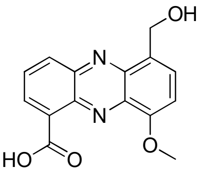

$C_{15}H_{12}N_2O_4$
Exact mass:284.0797
Griseoluteic acid
(2)

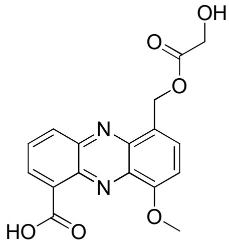

$C_{17}H_{14}N_2O_6$
Exact mass:342.0852
Griseolutein A
(3)

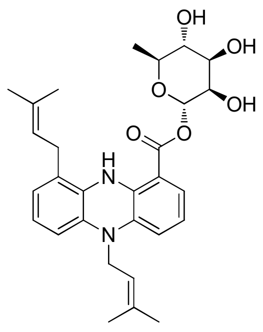

$C_{29}H_{36}N_2O_6$
Exact mass:508.2573
Aestivophoenin C
(4)

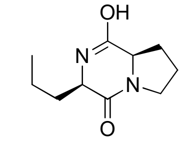

$C_{11}H_{18}N_2O_2$
Exact mass:210.1368
cyclo(D)-Pro-(D)-Leu
(5)

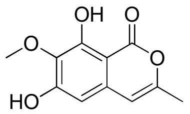

C11H10O5
Exact mass:222.0528
Reticulol
(6)

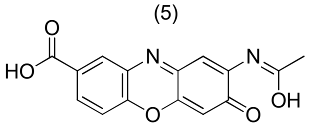

C15H10N2O5
Exact mass:298.0590
Umycin-A
(7)

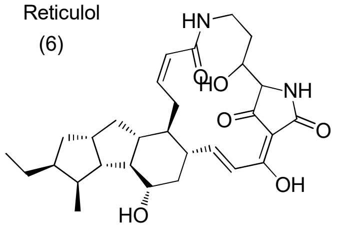

$C_{29}H_{40}N_2O_6$
Exact mass:512.2886
Dihydromaltophilin
(8)

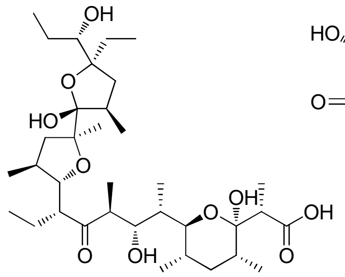

$C_{34}H_{60}O_{10}$
Exact mass:628.4186
Ferensimycin-A
(9)

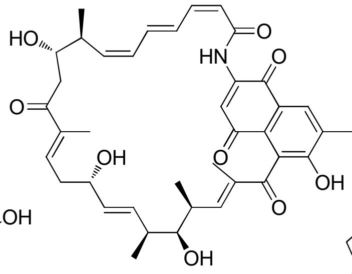

$C_{39}H_{45}NO_9$
Exact mass:671.3094
Naphthomycin C
(10)

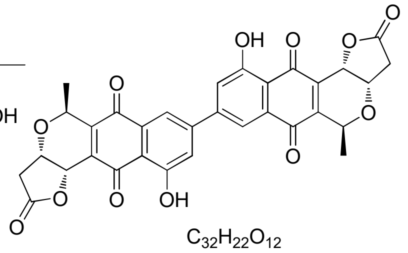

$C_{32}H_{22}O_{12}$
Exact mass:598.1111
Crisamicin A
(11)

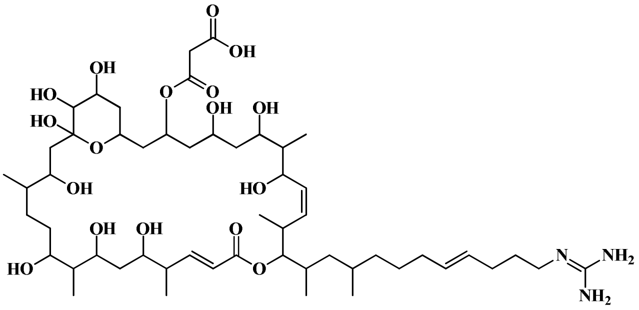

$C_{53}H_{93}N_3O_{17}$

Exact mass:1043.6505

Neocopiamycin A

12

Fig S30. Dereplicated metabolites from metabolomic analysis of. *Micromonospora* sp UR 56

Table (S2). The dereplication results of the ethyl acetate fraction of *Actinokinospora* EG49.

Polarity	Rt(min)	m/z	Formula	Name	Source	Activity	Class	Reference
**$[M+H]^+$**	11.98	611.2127	$C_{32}H_{34}O_{12}$	Actinosporin A (13)	Actinokinospora EG 49	Anti-trypanosomal	Anthraquinone	[13]
**$[M+H]^+$**	10.60	519.1444	$C_{25}H_{26}O_{12}$	Actinosporin B (14)	Actinokinospora EG 49	---------------------	Anthraquinone	[13]
**$[M-H]^-$**	4.80	467.1350	$C_{25}H_{24}O_9$	Atramycin A (15)	Streptomyces atratus	Antitumor	Anthraquinone	[14]
**$[M-H]^-$**	4.35	451.1386	$C_{25}H_{24}O_8$	Atramycin B (16)	Streptomyces atratus	Antitumor	Anthraquinone	[14]
**$[M+H]^+$**	3.14	152.0709	$C_8H_9NO_2$	Streptokordin (17)	Actinobacteria Streptomyces sp	Cytotoxic	Methyl pyridine	[15]
**$[M+H]^+$**	2.43	197.1289	$C_{10}H_{16}N_2O_2$	Cyclo-(proly-valyl)(18)	Actinobacteria	----------------	diketopiperazine	
**$[M-H]^-$**	2.31	463.1708	$C_{22}H_{28}N_2O_9$	Antibiotic USF 19A(19)	Streptomyces sp. USF19	Lipoxygenase inhibitor	Quinone	[16]
**$[M+H]^+$**	12.65	701.481	$C_{38}H_{68}O_{11}$	Inostamycin (20)	Streptomyces sp.	Antibacterial and antimetastic	polyether	[17]

**[M-H]⁻**	6.87	253.049	$C_{15}H_{10}O_4$	Daidzein(21)	Micromonospora halophytica	Antioxidant with anticancer and bone-protectant	isoflavones	[18]
**[M-H]⁻**	4.90	341.0657	$C_{18}H_{14}O_7$	Momofulvenone-A(22)	Streptomyces diastatochromogenes (strain Tue 2895)	**------------------**	Quinone	[19]
**[M+H]⁺**	12.03	523.3602	$C_{26}H_{46}N_6O_5$	Lucentamycin C (23)	Actinobacteria Nocardiopsis lucentensis	cytotoxic	Peptide	[20]

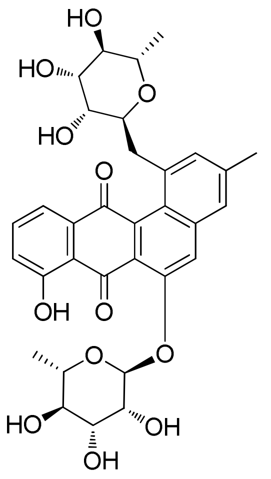

$C_{32}H_{34}O_{12}$
Exact mass:610.2050
Actinosporin A
(13)

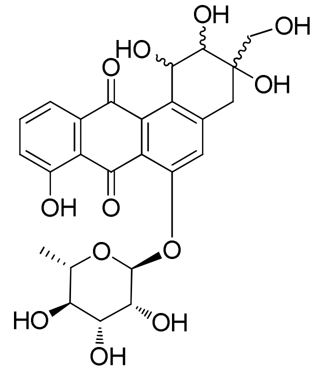

$C_{25}H_{26}O_{12}$
Exact mass:518.1424
Actinosporin B
(14)

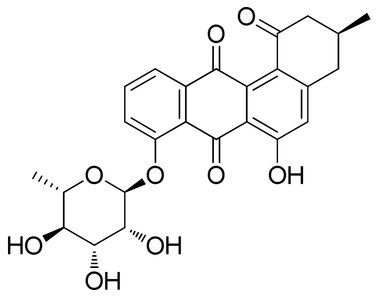

$C_{25}H_{24}O_{9}$
Exact mass:468.1420
Atramycin A
(15)

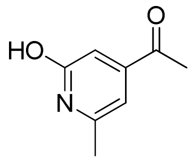

$C_{8}H_{9}NO_{2}$
Exact mass : 151.0633
streptokordin
(17)

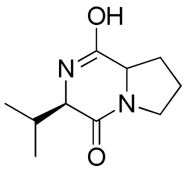

$C_{10}H_{16}N_{2}O_{2}$
Exact mass:196.1216
Cyclo-(proly-valyl)
(18)

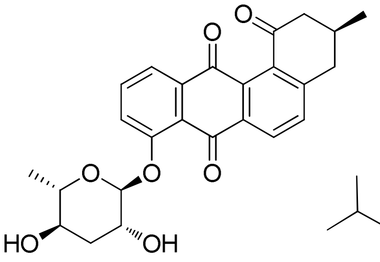

$C_{25}H_{24}O_{8}$
Exact mass:452.1471
Atramycin B
(16)

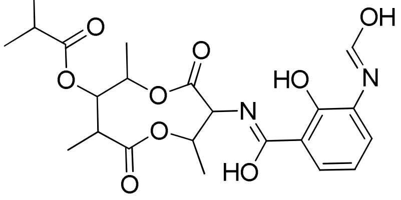

$C_{22}H_{28}N_{2}O_{9}$
Exact mass:464.1795
Antibiotic USF 19A
(19)

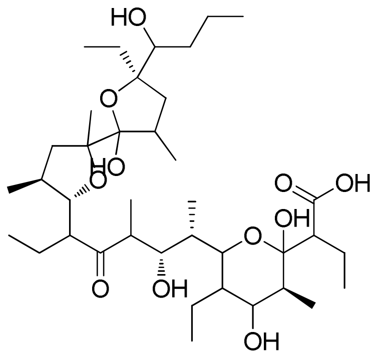

$C_{38}H_{68}O_{11}$
Exact mass:700.4762
Inostamycin
(20)

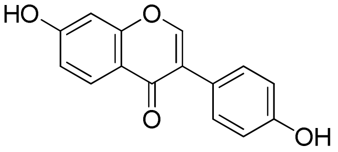

$C_{15}H_{10}O_{4}$
Exact mass:254.0579
Daidzein
(21)

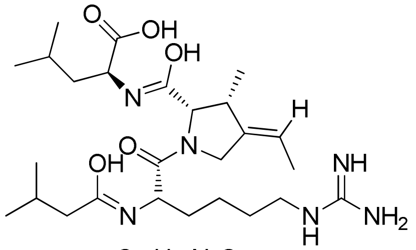

$C_{26}H_{46}N_{6}O_{5}$
Exact mass:522.3530
Lucentamycin C
(23)

Fig S31. Dereplicated metabolites from metabolomic analysis of *Actinokineospora sp. EG49*.

Table (S3).The dereplication results of the ethyl acetate fraction of co-culture.

Name	Formula	m/z	Source	Class	Activity	Reference
**Phenazine-1-carboxylic acid (24) Tubermycin B**	$C_{13}H_8N_2O_2$	224.0580	Streptomyces antibioticus strain Tue 2706	Phenazine	Antibacterial	[21]
Saphenic acid methyl ether (methyl saphenate)(25)	$C_{16}H_{14}N_2O_3$	282.0999	Streptomyces antibioticus strain Tue 2706	Phenazine	--------------------	[22]
**dimethyl Phenazine 1,6–dicarboxylate (26)**	$C_{16}H_{12}N_2O_4$	296.0781	Prod. by a Streptomyces sp	phenazine	--------------------	[23]
**Phenazine -1,6-carboxylic acid mono methyl ester (phencomycin)(27)**	$C_{15}H_{10}N_2O_4$	282.0648	Prod. by a Streptomyces sp	Phenazine	Antibacterial and antitumor	[23]
**Citreamicin ϵ (28)**	$C_{30}H_{25}NO_{11}$	575.14275	Actinobacteria Streptomyces caelestis	Quinone	Antibacterial including MRSA	[24]
**Antibiotic SF 2446A2(29)**	$C_{34}H_{35}NO_{15}$	697.2001	Streptomyces sp. sf2446	Quinone	Active against Gram-positive bacteria and mycoplasmas	[25]
**7 -Hydroxybenanomicin-A (30)**	$C_{39}H_{41}NO_{20}$	843.2216	Actinomadura sp. mh193-16f4	Quinone	-------------------	[26]
**11-Hydroxycinerubin A (31)**	$C_{42}H_{53}NO_{17}$	843.3308	Streptomyces galilaeus	Quinone	--------------------	[27]
**Tetrin B (32)**	$C_{34}H_{51}NO_{14}$	697.3304	Streptomyces sp.	Macrolide	Antifungal	[28]
**Maridomycin-II (33)**	$C_{42}H_{69}NO_{16}$	843.4610	Streptomyces platensis-malvinus	Macrolide	Antibacterial	[29]
**Dactylocycline-A(34)**	$C_{31}H_{40}ClN_3O_{13}$	697.2244	Dactylosporangium sp.	Tetracyclin	Antibacterial	[30]

			(ATCC 53693)			
**pulicatin A (35)**	$C_{11}H_{13}NO_2S$	223.0667	Actinobacteria Streptomyces sp	Thiazole	-------------------	[31]
**Antibiotic MSD 92 (36)**	$C_8H_9N_5O_3$	223.0699	Streptomyces sp. IM2096<br><br>Streptomyces sp. GW7/2495	pyrimidotriazine	Antibacterial	[32]
**MKN-003A (37)**	$C_{13}H_{20}O_3$	224.1412	Actinobacteria Streptomyces sp	butenolide	Antifouling	[33]
**Cairomycin B (38)**	$C_{10}H_{15}N_3O_3$	225.1108	Streptomyces AS-C-19	Cyclic peptide	Antibacterial	[34]
**(E)-4-Phenyl-3-(pyridine-2-yl)but-2-en-1-ol (39)**	$C_{15}H_{15}NO$	225.1148	Streptomyces sp.	Pyridine	Antiproliferative agent	[35]
**Lemonomycin (40)**	$C_{27}H_{41}N_3O_9$	551.2837	Streptomyces candidus	isoquinoline	Antibacterial and antitumor	[36]
**Sinefungin VA (41)**	$C_{23}H_{37}N_9O_7$	551.2810	Streptomyces sp. K05-0178	nucleoside	antitrypansomal	[37]
**Deacetylanisomycin (42)**	$C_{12}H_{17}NO_3$	223.1203	marine Streptomyces sp. SA3097	Pyrrolidine	---------------------	[38]
**1-Hydroxyanthraquinone (43)**	$C_{14}H_8O_3$	224.0468	Synthetic	Quinone	---------------------	[39]
**Pyramidamycin D (44)**	$C_{10}H_{12}N_2O_4$	224.0791	Streptoverticillium morookaense	Amide	----------------------	[40]
**5-Chloro-6-methoxy-1-methylisatin (45)**	$C_{10}H_8ClNO_3$	225.0187	Micromonospora carbonacea	Indole	-----------------------	[41]

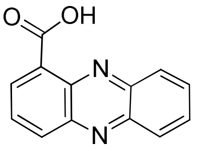

$C_{13}H_8N_2O_2$
Exact mass:224.0586
Phenazine-1-carboxylic acid
(24)

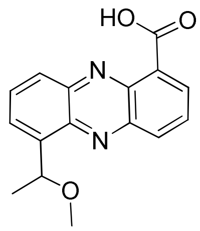

$C_{16}H_{14}N_2O_3$
Exact mass:282.1004
Methyl saphenate
(25)

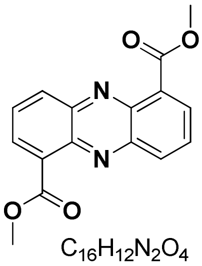

$C_{16}H_{12}N_2O_4$
Exact mass:296.0797
Dimethyl phenazine-1,6-dicarboxylate
(26)

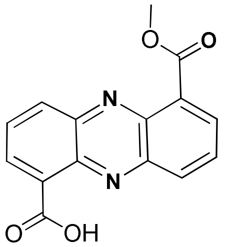

$C_{15}H_{10}N_2O_4$
Exact mass:282.0641
phencomycin
(27)

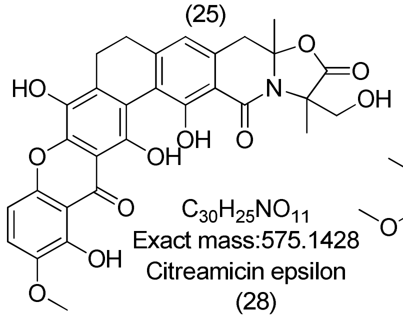

$C_{30}H_{25}NO_{11}$
Exact mass:575.1428
Citreamicin epsilon
(28)

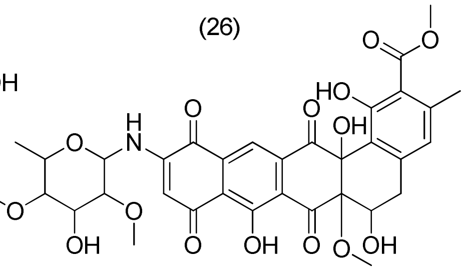

$C_{34}H_{35}NO_{15}$
Exact mass:697.2007
SF 2446A2
(29)

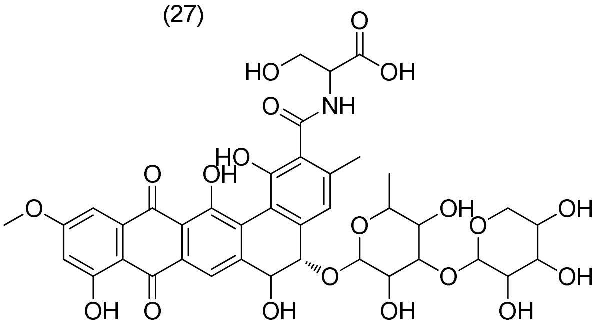

$C_{39}H_{41}NO_{20}$
Exact mass:843.2222
7-Hydroxybenanomicin-A
(30)

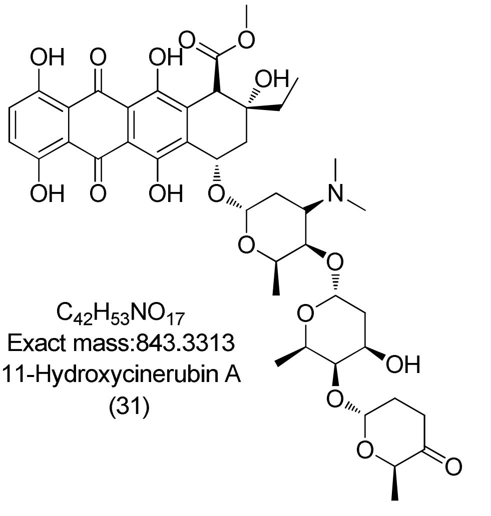

$C_{42}H_{53}NO_{17}$
Exact mass:843.3313
11-Hydroxycinerubin A
(31)

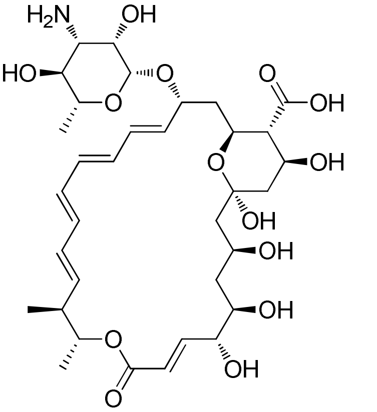

$C_{34}H_{51}NO_{14}$
Exact mass:697.3310
Tetrin B (32)

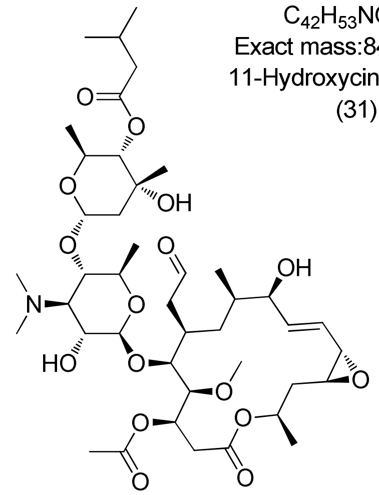

$C_{42}H_{69}NO_{16}$
Exact mass:843.4616
Maridomycin-II
(33)

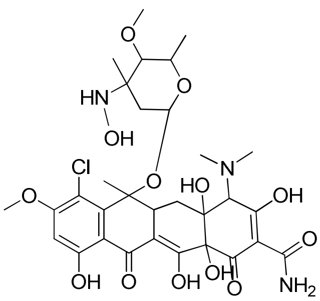

$C_{31}H_{40}ClN_3O_{13}$
Exact mass:697.2250
Dactylocycline-A
(34)

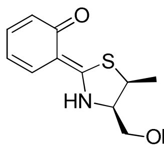

$C_{11}H_{13}NO_2S$
Exact mass:223.0667
pulicatin A
(35)

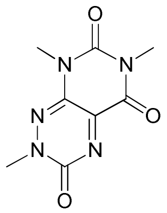

$C_8H_9N_5O_3$
Exact mass:223.0705
Antibiotic MSD 92
(36)

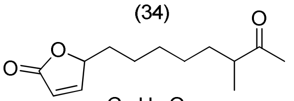

$C_{13}H_{20}O_3$
Exact mass:224.1412
MKN-003A
(37)

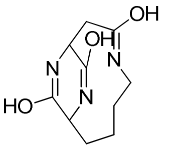

$C_{10}H_{15}N_3O_3$
Exact mass:225.1113
Cairomycin B
(38)

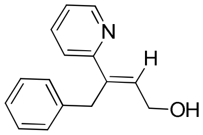

$C_{15}H_{15}NO$
Exact mass:225.1154
E)-4-Phenyl-3-(pyridine-2-yl)but-2-en-1-ol
(39)

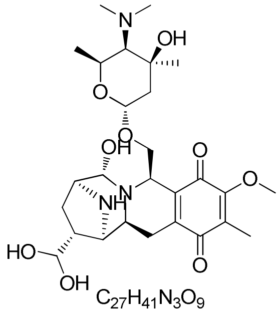

$C_{27}H_{41}N_3O_9$
Exact mass:551.2843
Lemonomycin
(40)

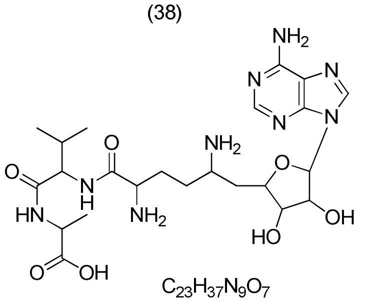

$C_{23}H_{37}N_9O_7$
Exact mass:551.2817
Sinefungin VA
(41)

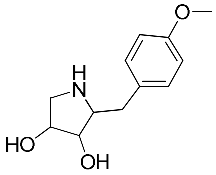

$C_{12}H_{17}NO_3$
Exact mass:223.1208
Deacetylanisomycin
(42)

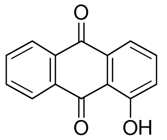

$C_{14}H_8O_3$
Exact mass:224.0473
1-Hydroxyanthraquinone
(43)

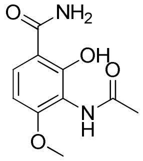

$C_{10}H_{12}N_2O_4$
Exact mass:224.0797
Pyramidamycin D
(44)

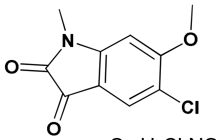

$C_{10}H_8CLNO_3$
Exact mass:225.0193
5-Chloro-6-methoxy-1-methyl-1H-indole-2,3-dione
(45)

Fig S32. Dereplicated metabolites from metabolomic analysis of *co-culture Micromonospora* sp. UR 56 and Actinokineospora *sp. EG49*.

1. Byng, G.S.; Turner, J.M. Phenazine biosynthesis by a pseudomonad 1975.

2. Wang, Y.; Luo, Q.; Zhang, X.; Wang, W. Isolation and purification of a modified phenazine, griseoluteic acid, produced by Streptomyces griseoluteus P510. *Res. Microbiol.* **2011**, *162*, 311–319.

3. Challand, S.R.; Herbert, R.B.; Holliman, F.G. A new phenazine synthesis. The synthesis of griseoluteic acid, griseolutein A, and methyl diacetylgriseolutein B. *J. Chem. Soc. D Chem. Commun.* **1970**, 1423–1425.

4. Kunigami, T.; Shin-Ya, K.; Furihata, K.; Furihata, K.; Hayakawa, Y.; Seto, H. A novel neuronal cell protecting substance, aestivophoenin C, produced by Streptomyces purpeofuscus. *J. Antibiot. (Tokyo).* **1998**, *51*, 880–882.

5. Fdhila, F.; Vázquez, V.; Sánchez, J.L.; Riguera, R. dd-Diketopiperazines: Antibiotics Active against Vibrio a nguillarum Isolated from Marine Bacteria Associated with Cultures of Pecten m aximus. *J. Nat. Prod.* **2003**, *66*, 1299–1301.

6. Mitscher, L.A.; Andres, W.W.; McCrae, W. Reticulol, a new metabolic isocoumarin. *Experientia* **1964**, *20*, 258–259.

7. Abdelfattah, M.S. A new bioactive aminophenoxazinone alkaloid from a marine-derived actinomycete. *Nat. Prod. Res.* **2013**, *27*, 2126–2131.

8. Graupner, P.R.; Thornburgh, S.; Mathieson, J.T.; Chapin, E.L.; Kemmitt, G.M.; Brown, J.M.; Snipes, C.E. Dihydromaltophilin; a novel fungicidal tetramic acid containing metabolite from Streptomyces sp. *J. Antibiot. (Tokyo).* **1997**, *50*, 1014–1019.

9. Kusakabe, Y.; Mizuno, T.; Kawabata, S.; Tanji, S.; Seino, A.; Seto, H.; Take, N. Ferensimycins A and B, two polyether antibiotics. *J. Antibiot. (Tokyo).* **1982**, *35*, 1119–1129.

10. Mochizuki, J.; Kobayashi, E.; Furihata, K.; Kawaguchi, A.; Seto, H.; Take, N. New ansamycin antibiotics, naphthoquinomycins A and B, inhibitors of fatty acid synthesis in Escherichia coli. *J. Antibiot. (Tokyo).* **1986**, *39*, 157–161.

11. Arai, T.; Uno, J.U.N.; Horimi, I.; Fukushima, K. Isolation of neocopiamycin A from Streptomyces hygroscopicus var. crystallogenes, the copiamycin source. *J. Antibiot. (Tokyo).* **1984**, *37*, 103–109.

12. Nelson, R.A.; Pope Jr, J.A.; Luedemann, G.M.; Mcdaniel, L.E.; Schaffner, C.P. Crisamicin A, A new antibiotic from micromonospora. *J. Antibiot. (Tokyo).* **1986**, *39*, 335–344.

13. Abdelmohsen, U.; Cheng, C.; Viegelmann, C.; Zhang, T.; Grkovic, T.; Ahmed, S.; Quinn, R.; Hentschel, U.; Edrada-Ebel, R. Dereplication strategies for targeted isolation of new antitrypanosomal actinosporins A and B from a marine sponge associated-Actinokineospora sp. EG49. *Mar. Drugs* **2014**, *12*, 1220–1244.

14. Fujioka, K.; Furihata, K.; Shimazu, A.; Hayakawa, Y.; Seto, H. Isolation and characterization of atramycin A and atramycin B, new isotetracenone type antitumor antibiotics. *J. Antibiot. (Tokyo).* **1991**, *44*, 1025–1028.

15. Jeong, S.-Y.; Shin, H.J.; Kim, T.S.; Lee, H.-S.; Park, S.; Kim, H.M. Streptokordin, a new cytotoxic compound of the methylpyridine class from a marine-derived Streptomyces sp. KORDI-3238. *J. Antibiot. (Tokyo).* **2006**, *59*, 234–240.

16. Komoda, T.; Morimitsu, Y.; Hirota, H.; Hirota, A. USF-19A, a new lipoxygenase inhibitor from Streptomyces sp. *Biosci. Biotechnol. Biochem.* **1995**, *59*, 924–926.

17. Imoto, M.; Umezawa, K.; Takahashi, Y.; Naganawa, H.; Iitaka, Y.; Nakamura, H.; Koizumi, Y.; Sasaki, Y.; Hamada, M.; Sawa, T. Isolation and structure determination of inostamycin, a novel inhibitor of phosphatidylinositol turnover. *J. Nat. Prod.* **1990**, *53*, 825–829.

18. Hirakura, K.; Morita, M.; Nakajima, K.; Sugama, K.; Takagi, K.; Niitsu, K.; Ikeya, Y.; Maruno, M.; Okada, M. Phenolic glucosides from the root of Pueraria lobata. *Phytochemistry* **1997**, *46*, 921–928.

19. Volkmann, C.; Rössner, E.; Metzler, M.; Zähner, H.; Zeeck, A. Metabolic products of microorganisms, 271. Momofulvenone A and B, new benzo [b] fluorene quinones from Streptomyces. *Liebigs Ann.* **1995**, *1995*, 1169–1172.

20. Cho, J.Y.; Williams, P.G.; Kwon, H.C.; Jensen, P.R.; Fenical, W. Lucentamycins A− D, cytotoxic peptides from the marine-derived actinomycete Nocardiopsis lucentensis. *J. Nat. Prod.* **2007**, *70*, 1321–1328.

21. Jayatilake, G.S.; Thornton, M.P.; Leonard, A.C.; Grimwade, J.E.; Baker, B.J. Metabolites from an Antarctic sponge-associated bacterium, Pseudomonas aeruginosa. *J. Nat. Prod.* **1996**, *59*, 293–296.

22. Geiger, A.; Keller-Schierlein, W.; Brandl, M.; ZÄHNER, H. Metabolites of microorganisms. 247 phenazines from Streptomyces antibioticus, strain Tü 2706. *J. Antibiot. (Tokyo).* **1988**, *41*, 1542–1551.

23. Chatterjee, S.; Vijayakumar, E.K.S.; Franco, C.M.M.; Maurya, R.; Blumbach, J.; Ganguli, B.N. Phencomycin, a new antibiotic from a Streptomyces species HIL Y-9031725. *J. Antibiot. (Tokyo).* **1995**, *48*, 1353–1354.

24. Hopp, D.C.; Milanowski, D.J.; Rhea, J.; Jacobsen, D.; Rabenstein, J.; Smith, C.; Romari, K.; Clarke, M.; Francis, L.; Irigoyen, M. Citreamicins with potent gram-positive activity. *J. Nat. Prod.* **2008**, *71*, 2032–2035.

25. Takeda, U.; Okada, T.; Takagi, M.; Gomi, S.; Itoh, J.; Sezaki, M.; Ito, M.; MIYADOH, S.; SHOMURA, T. SF2446, NEW BENZO [α] NAPHTHACENE QUINONE ANTIBIOTICS. *J. Antibiot. (Tokyo).* **1988**, *41*, 417–424.

26. Kondo, S.; Gomi, S.; Uotani, K.; Miyakawa, S.; Inouye, S.; Ikeda, D.; Takeuchi, T. New hydroxybenanomicins produced by Actinomadura. *Drugs Exp. Clin. Res.* **1992**, *18*, 217–224.

27. Oki, T.; Yoshimoto, A.; Matsuzawa, Y.; Takeuchi, T.; Umezawa, H. Biosynthesis of anthracycline antibiotics by Streptomyces galilaeus. *J. Antibiot. (Tokyo).* **1980**, *33*, 1331–1340.

28. Rinehart Jr, K.L.; Tucker, W.P.; Pandey, R.C. 1 Polyene antibiotics. III. Structure of tetrin B. *J. Am. Chem. Soc.* **1971**, *93*, 3747–3751.

29. Muroi, M.; IZAWA, M.; ASAI, M.; KISHI, T.; MIZUNO, K. MARIDOMYCIN, A NEW MACROLIDE ANTIBIOTIC. II. *J. Antibiot. (Tokyo).* **1973**, *26*, 199–205.

30. Tymiak, A.A.; Aklonis, C.; Bolgar, M.S.; Kahle, A.D.; Kirsch, D.R.; O'Sullivan, J.; Porubcan, M.A.; Principe, P.; Trejo, W.H. Dactylocyclines: novel tetracycline glycosides active against tetracycline-resistant bacteria. *J. Org. Chem.* **1993**, *58*, 535–537.

31. Lin, Z.; Antemano, R.R.; Hughen, R.W.; Tianero, M.D.B.; Peraud, O.; Haygood, M.G.; Concepcion, G.P.; Olivera, B.M.; Light, A.; Schmidt, E.W. Pulicatins A− E, neuroactive thiazoline metabolites from cone snail-associated bacteria. *J. Nat. Prod.* **2010**, *73*, 1922–1926.

32. Wang, H.; Lim, K.L.; Yeo, S.L.; Xu, X.; Sim, M.M.; Ting, A.E.; Wang, Y.; Yee, S.; Tan, Y.H.; Pallen, C.J. Isolation of a novel protein tyrosine phosphatase inhibitor, 2-methyl-fervenulone, and its precursors from Streptomyces. *J. Nat. Prod.* **2000**, *63*, 1641–1646.

33. Strand, M.; Carlsson, M.; Uvell, H.; Islam, K.; Edlund, K.; Cullman, I.; Altermark, B.; Mei, Y.-F.; Elofsson, M.; Willassen, N.-P. Isolation and characterization of anti-adenoviral secondary metabolites from marine actinobacteria. *Mar. Drugs* **2014**, *12*, 799–821.

34. Shimi, I.R.; Abedallah, N.; Fathy, S. Cairomycin B, a new antibiotic. *Antimicrob. Agents Chemother.* **1977**, *11*, 373–375.

35. Shin, C.; Lim, H.; Moon, S.; Kim, S.; Yong, Y.; Kim, B.-J.; Lee, C.-H.; Lim, Y. A novel antiproliferative agent, phenylpyridineylbutenol, isolated from Streptomyces sp. *Bioorg. Med. Chem. Lett.* **2006**, *16*, 5643–5645.

36. He, H.; Shen, B.; Carter, G.T. Structural elucidation of lemonomycin, a potent antibiotic from Streptomyces candidus. *Tetrahedron Lett.* **2000**, *41*, 2067–2071.

37. Niitsuma, M.; Hashida, J.; Iwatsuki, M.; Mori, M.; Ishiyama, A.; Namatame, M.; Nishihara-Tsukashima, A.; Matsumoto, A.; Takahashi, Y.; Yamada, H. Sinefungin VA and dehydrosinefungin V, new antitrypanosomal antibiotics produced by Streptomyces sp. K05-0178. *J. Antibiot. (Tokyo).* **2010**, *63*, 673–679.

38. Hosoya, Y.; Kameyama, T.; Naganawa, H.; Okami, Y.; Takeuchi, T. Anisomycin and new congeners active against human tumor cell lines. *J. Antibiot. (Tokyo).* **1993**, *46*, 1300–1302.

39. Khanapure, S.P.; Reddy, R.T.; Biehl, E.R. The preparation of anthraquinones and anthracyclinones via the reaction of haloarenes and cyanophthalides under aryne-forming conditions. *J. Org. Chem.* **1987**, *52*, 5685–5690.

40. Shaaban, K.A.; Shepherd, M.D.; Ahmed, T.A.; Nybo, S.E.; Leggas, M.; Rohr, J. Pyramidamycins AD and 3-hydroxyquinoline-2-carboxamide; cytotoxic benzamides from Streptomyces sp. DGC1. *J. Antibiot. (Tokyo).* **2012**, *65*, 615–622.

41. Omote, Y.; Tazawa, H.; Fujinuma, Y.; Sugiyama, N. Synthesis of 5-Chloro-6-Methoxy-N-Methylisatin. *Bull. Chem. Soc. Jpn.* **1969**, *42*, 3016–3017.
